# Aridification and major geotectonic landscape change shaped an extraordinary species radiation across a world’s extreme elevational gradient

**DOI:** 10.1038/s42003-024-07181-7

**Published:** 2024-11-13

**Authors:** Adrián Villastrigo, Steven J. B. Cooper, Barbara Langille, Erinn P. Fagan-Jeffries, William F. Humphreys, Lars Hendrich, Michael Balke

**Affiliations:** 1 Division of Entomology, SNSB-Zoologische Staatssammlung München, Munich, Germany; 2 South Australian Museum, Adelaide, South Australia Australia; 3 Department of Ecology and Evolutionary Biology, School of Biological Sciences, and Environment Institute, The University of Adelaide, Adelaide, South Australia Australia; 4 Western Australian Museum, Welshpool DC, Western Australia Australia; 5 School of Biological Sciences, University of Western Australia, Perth, Western Australia Australia; 6 GeoBioCenter, Ludwig Maximilians University, Munich, Germany

**Keywords:** Speciation, Evolutionary ecology, Entomology

## Abstract

Understanding the profound influence of climatic and tectonic histories on adaptation and speciation is a crucial focus in biology research. While voyages like Humboldt’s expedition shaped our understanding of adaptation, the origin of current biodiversity remains unclear – whether it arose in situ or through dispersal from analogous habitats. Situated in the geologically complex Australopacific region, our study focuses on *Limbodessus* diving beetles (Dytiscidae), a diverse genus distributed from underground aquifers in Western Australia to alpine meadows in New Guinea. Using low-coverage whole-genome sequencing, we established a time-calibrated phylogenetic tree, elucidating *Limbodessus*’ origin in the mid-late Miocene, most likely in the Sahul continent (i.e., Australia and New Guinea) and western Pacific archipelagos. Our results provide evidence for parallel colonization and speciation at extreme altitudinal ends, driven by aridification in Australia, influencing subterranean colonization, and in situ diversification of alpine taxa by passive-uplifting of local biota in New Guinea. Furthermore, our findings highlight instances of subterranean speciation in isolated underground aquifers, marked by recurrent independent colonizations of this habitat.

## Abstract (web summary)

This study on *Limbodessus* diving beetles reveals a mid-late Miocene origin in the Sahul region, with arid-driven subterranean speciation in Australia and alpine diversification in New Guinea through passive uplift.

## Introduction

Mountain ecosystems have captivated naturalists for over 200 years^1^, influencing a wide range of disciplines, including evolution, ecology and biogeography. Alexander von Humboldt’s *Tableau Physique*^2^, depicting plant communities along a schematic elevational gradient, marked an early attempt to comprehend the vertical zonation of biota^3^. Modern studies of altitudinal gradients often integrate the reconstruction of evolutionary histories, where physical habitat characteristics impose significant constraints on species distribution and adaptation^4,5^. These constraints, mutable and influenced by changing environmental conditions on a macroevolutionary scale^6^, shape both current and past patterns of biodiversity. When these constraints are relaxed or extended, they create “ecological opportunities”^7^ that catalyze the diversification and emergence of novel species, thereby enhancing the overall complexity and richness of biodiversity. Such ecological opportunities are frequent in regions with exceptional tectonic activity, where the physical settings are altered by the emergence of new habitats (e.g., newly formed islands^8^) or the transformation of existing habitats (e.g., via the emergence of mountain ranges^9^). These environmental changes contribute to the development of a rich taxonomic diversity and diverse ecological communities^10^. However, it remains uncertain whether mountain biodiversity and their distribution along altitudinal gradients, can be attributed to in situ diversification, resulting from ecological opportunities for a preexisting biota through passive uplifting^11^ or whether it is the result of dispersal from analogous or surrounding habitats. While the latter is commonly accepted as the standard evolutionary paradigm, it overlooks the potential effect of pre-existing species and their evolution during orogeny (i.e., mountain formation resulting from tectonic uplift^12^).

An extraordinary example of an area that combines both ancient and recently formed, tectonically active features is the Oceania region, renowned for hosting some of the world’s most biologically diverse and geologically complex archipelagos^13,14^. This region encompasses diverse landmasses that were once part of the Sahul shelf (hereafter referred to as Sahul), plus it features a myriad of archipelagos of varying ages, spanning from New Caledonia to French Polynesia^15^. Sahul comprises two markedly different landmasses: New Guinea, the largest and highest tropical island^16^, and Australia, the driest continent on Earth^17^. New Guinea, one of the world’s most complex geotectonic islands^18^, emerged from the collision of the Australian and Pacific plates and has undergone significant landscape changes since the mid-late Miocene^19^. During this transformative period, the ocean floor was unfolded to an altitude of approximately 5000 m, giving rise to vast tropical upper montane and alpine ecosystems, often referred to as sky islands^20,21^. These ecosystems, surrounded by tropical lowlands, foster exceptional biodiversity and local endemism across diverse biomes characterized by extreme altitudinal and climatic gradients^22^. The presence of emerged isolated terrains in present-day New Guinea is considered to have started in the Miocene^19^, though recent studies suggest a more ancient origin, with some narrow terrestrial areas emerging since the Oligocene^23^ and acting as important diversification pumps^24^. In contrast, Australia has remained relatively stable in terms of both size and altitude since the Paleocene^25^, notwithstanding extensive aridification from the mid-late Miocene onward, with a brief return to wet conditions within the Pliocene, followed by a further period of aridity during the Pleistocene^26^. This environmental shift led to the generation of subterranean island-like calcrete (carbonate) aquifers in the arid zone of Australia, which harbor highly diverse subterranean ecosystems^27^. The parallel existence of island-like habitats at both extremes of the elevational gradient presents an intriguing scenario, offering unique environmental conditions that provide new niches for taxa to exploit. Therefore, this region provides an ideal system for studying contrasting environmental pressures, wherein habitat formation and modifications generate selection pressures that may open new opportunities to species, including habitat specialization and the promotion of a distinct biodiversity^11,28^. Previous studies have explored how orogeny and environmental change have impacted the diversification of species within New Guinea^14,20,29–31^ and the broader Sahul region^16,32^. The study of rodents by Roycroft et al.^16^ explored diversification across all major biomes associated with Sahul, including oceanic islands, however, there is a notable gap in the literature exploring adaptive radiations at the extreme opposing ends of the altitudinal gradient, encompassing alpine and subterranean taxa.

Our study aims to determine how the climatic and tectonic histories, via extreme aridification and orogeny, shaped the evolution of the unique biota of Australia and New Guinea. This is achieved by reconstructing the evolutionary history of *Limbodessus* diving beetles (family Dytiscidae) that are found throughout the region from lowlands and underground aquifers in Australia up to elevations of 4000 m in the subalpine meadows of New Guinea, showcasing multiple biome transitions^33^. *Limbodessus* stands out as the most speciose genus of subterranean diving beetles in the world^34,35^, but also includes a widespread species in Australia and New Guinea with its range expanding as far as Thailand and Japan in the west. We reconstructed the biogeographic and evolutionary history of these beetles, focusing on their adaptation to different habitats along the altitudinal gradient. We employed low-coverage whole-genome sequencing data and museum specimens to construct a calibrated phylogenomic tree. Ancestral area and trait reconstructions were applied to understand how frequently and when *Limbodessus* reached opposite altitudinal extremes and to link this to major geotectonic and climate change events. Our results indicated that *Limbodessus* likely originated in the mid-late Miocene within the Sahul continent. Subsequently, the genus exhibited parallel colonization of both the extreme altitudinal and ecomorphological gradients, as a result of constraint forces: (1) the reduction in surface water availability during Australian aridification, which led to frequent independent colonization and speciation within isolated calcrete aquifers (i.e., the Climatic Relict Hypothesis^36^); (2) passive uplift and in situ speciation in the New Guinea orogen.

## Results and discussion

### Taxon sampling and phylogenetic analyses

**Fig. 1:**
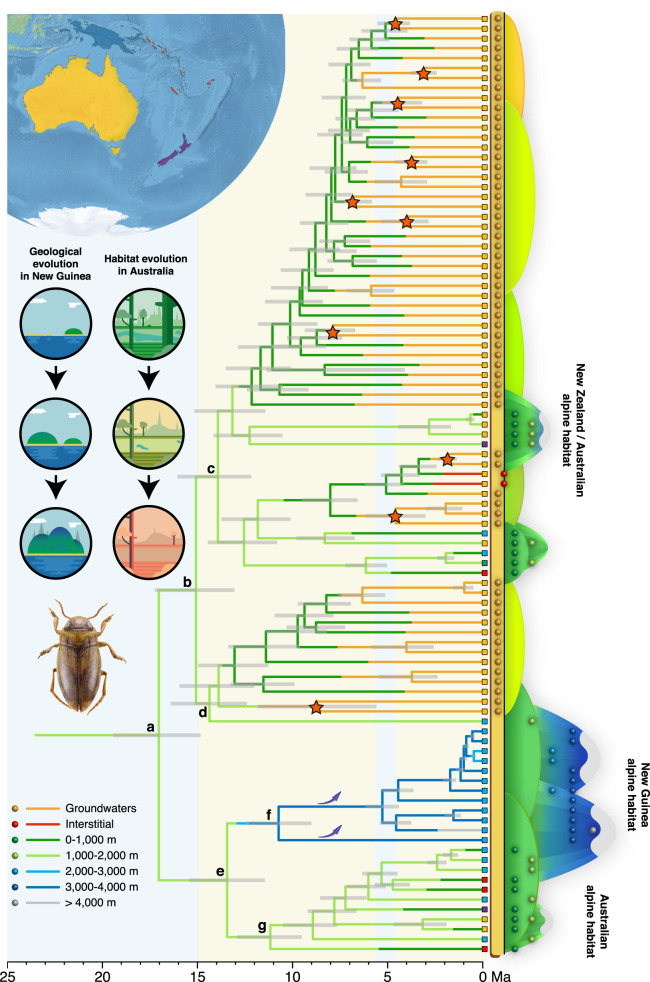
BEAST maximum clade credibility tree for *Limbodessus* showing ancestral habitat and maximum altitude evolution. Branch colors represent the maximum altitude that each taxon could reach, with terminal branches displaying habitat evolution (i.e., groundwaters, interstitial, or epigean). Colored squares on leaves depict geographical areas as in the top-left corner map, except for the green color that represents a wide geographic range including all areas. Colored circles in the right diagram represent different altitudinal bands at which each species is found. Blue arrows represent passive-uplifting processes, and orange stars on nodes represent subterranean speciation within isolated aquifers. Habitus photograph correspond to *Limbodessus baliem* Balke & Hendrich 2015.

The molecular dataset for the diving beetle family (Dytiscidae) used in the Bayesian phylogenetic analyses comprised 357 ultraconserved elements (UCEs) from representatives of 41 genera. The preferred analysis provided a robust and well-supported topology for 37 out of 41 nodes (Supplementary Information, Table S1, Fig. S1), including a fully resolved topology for the tribe Bidessini, in which *Limbodessus* is included. This reconstruction served as a foundational framework for estimating crown ages within the subtribe Bidessini (Table 1) for use as secondary calibration points for the Bayesian analyses of *Limbodessus*. The dataset for ancestral state optimization of *Limbodessus* combined UCEs, as well as 26 additional markers (mitochondrial genomes and nuclear genes: rRNA operon, histones, arginine kinase, topoisomerase, and wingless) and included all but four described *Limbodessus* species plus some undescribed taxa (95 species in total) and 10 outgroups (Supplementary Information, Table S2). The preferred topology, which is strongly supported (Fig. 1 and Supplementary Information, Fig. S2), is notably superior to previous mitochondrial analyses of the genus^37,38^ and sets the temporal framework for further analyses. It resolves, for the first time, a key ancestral topological split delineating two clades: one clade predominantly comprises Australian species (Fig. 1, clade “b”), while the other includes the majority of New Guinean and Pacific species (Fig. 1, clades “f” and “g” respectively).

**Table 1:** Secondary calibration points extracted from the calibrated Dytiscidae analysis

Crown group	Age	Prior	Parameters
*Limbodessus*	15.79 (11.3–20.44)	Lognormal	x͂ = 15.79; SD = 2.9
*Limbodessus* + *Allodessus*	26.68 (21.62–32.36)	Lognormal	x͂ = 26.68; SD = 3.3
*Bidessini*	58.49 (51.81 –65.65)	Lognormal	x͂ = 58.49; SD = 4.2

### Node age estimation and biogeographic history

**Fig. 2:**
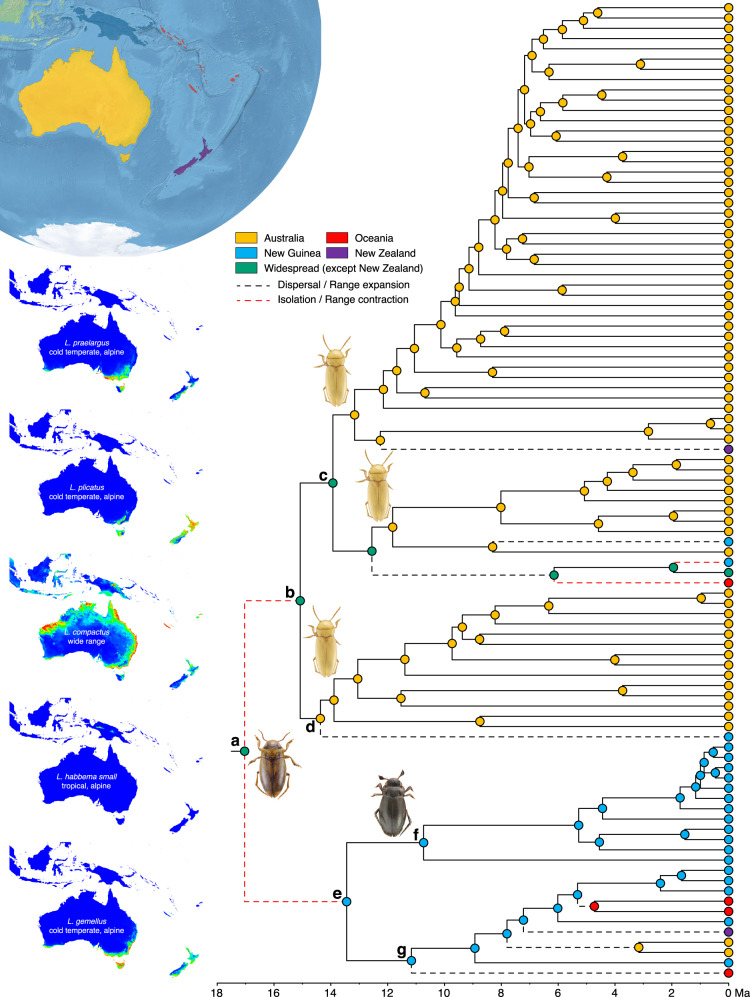
Ancestral area estimation for Limbodessus over the maximum clade credibility tree as inferred by BioGeoBears considering the preferred hypotheses (M1) and model that fits best our dataset (DEC + J). Left insets display ecological niche evolution for different species preferences. Habitus photographs correspond to *Limbodessus baliem* Balke & Hendrich, 2015 (ancestral node), *Limbodessus windarraensis* (Watts & Humphreys, 1999) (within clades “c” and “d”) and *Limbodessus alexanderi* Balke & Hendrich, 2015 (clade “f”).

The crown group origin of *Limbodessus* was estimated at the mid-late Miocene, c. 17 Ma [95% highest probability density (HPD): 14.83–19.41] (Figs. 1 and 2, clade “a”). Despite the precise node age estimation, the ancestral area estimation remains ambiguous, leaning towards a widespread ancestor in the area, including Australia and New Guinea (Sahul) but excluding New Zealand under the preferred hypothesis and model (M1, DEC + *J*, Table 2, Fig. 2, Supplementary Information, Fig. S3). Nonetheless, the high diversity in Sahul compared to its surroundings suggest it is the likely center of origin (Supplementary Information, Fig. S2). The contemporary New Guinea landmass originated from isolated islands, forming a proto-Papua archipelago, starting from either the Miocene^23^ or the Oligocene^13^. The collision between the Australian and Pacific plates triggered major geotectonic events, reshaping the regional landscape^19^. These transformative processes culminated in the establishment of the present-day New Guinea and a considerable orogeny, occurring during the past 8–5 Ma^15^. Similarly, the emergence of most Pacific archipelagos during the Pliocene^25^ further narrows the potential ancestral area to Sahul.

**Table 2:** Hypotheses tested and best model comparison

Hypotheses	Assumption	Best model	AICc	Root areas
M0	- Unguided analysis	DEC	128.5	BCDE
M1	- Standard origin of New Guinean - Custom dispersal matrix (standard)	DEC + *J*	116	ABCD
M2	- Standard origin of New Guinean - Custom dispersal matrix (standard) - *w* parameter	DEC + *J*	118.1	ABCD
M3	- Relaxed origin of New Guinean - Custom dispersal matrix (relaxed)	DEC	127	BCDE
M4	- Relaxed origin of New Guinea - Custom dispersal matrix (relaxed) - *w* parameter	DEC + *J*	118.1	ABCD

By standard origin, or New Guinea, we refer to the first emerged landmasses during the Miocene^19^, and the relaxed origin, or New Guinea, refers to the first emerged landmasses during the Oligocene^23^. Root areas represent the following states: East Asia (A), Australia (B), New Guinea (C), Pacific archipelagos (D), and New Zealand (E).

In accordance with these landscape changes, *Limbodessus* diversified into two main clades (Figs. 1 and 2). The first clade consists of 72 species, predominantly found in Australia (clade “b”). The second clade encompasses 21 species distributed across the Pacific, New Guinea, New Zealand, and two species in Australia (clade “e”). The origin of the latter was estimated to be New Guinea, specifically the proto-Papuan Archipelago, with a crown group age c. 13 Ma [95% HPD: 11.46–15.46]. The subsequent divergence within this clade gave rise to two subclades (clades “f” and “g”). Subclade “f” consists of microendemic species exclusive to upper montane to alpine habitats of New Guinea, situated at elevations ranging from c. 2800–4000 m high (*Alpine habitats*, see below). The other subclade (“g”) exhibits a highly heterogeneous geographical distribution, primarily inhabiting lower elevations in New Guinea (<2000 m). Notably, this subclade underwent back colonization to Australia as well as range expansion over the past c. 11 Ma [95% HPD: 9.52–12.92], reaching as far east as French Polynesia and extending to New Zealand. These range expansions across diverse regions suggest the presence of an ancestral species with great occasional dispersal capacity, followed by local segregation and speciation (*Colonization of Oceania*, see below).

Starting c. 15 M [95% HPD: 13.06–17.25], the mainly Australian clade (“b”) diversified across the continent, marked by at least four distinct range expansion events (Fig. 2): towards New Zealand, twice towards New Guinea, and an extensive spread across the entire Australopacific region and East Asia. Notably, the latter expansion, initiated at c. 12 Ma [95% HPD: 10.81–14.46], covered a vast geographic range and involved numerous biome transitions including temperate zones, subtropics and tropics, which are reflected in the ecological niche model (Fig. 2). Subsequently, between c. 2–6 Ma [95% HPD: 1.49–2.41 and 5.02–7.22], there were episodes of range contraction or isolation with speciation, resulting in two endemic species in New Guinea and Fiji.

The high diversity observed in Australia can be attributed to its evolutionary history: the Australian continent is an ancient landmass with a stable topography since the Paleocene^37^, with its last major marine intrusion c. 100 Ma^39^. The Australian Alps are also ancient formations that have existed for more than 100 Ma^40^. However, Australia has experienced large-scale aridification since the Miocene (c. 15 Ma). During this time, forests were gradually replaced by other vegetation forms, culminating in the spread of deserts that now cover a significant portion of the continent^41^. The consequential environmental changes prompted a profound adaptation among some aquatic fauna, compelling them to undergo a habitat transition into underground waters (*Life underground* section below).

### Ancestral altitude and habitat reconstructions

The evolution of altitudinal potential in *Limbodessus* was investigated considering the discrete ‘all-rates-different’ Markov model with five primary altitudinal states based on the maximum altitude at which species are found: below 1000 m; 1001–2000 m.; 2001–3000 m.; 3001–4000 m.; and above 4001 m. This scheme is subjective to some degree but it effectively captures major altitudinal bands that contain a significant amount of suitable habitat across the entire genus range (see *Materials and methods* section). Simultaneously, the evolution of habitat preference was reconstructed by considering three fundamental states: underground, interstitial, and epigean (surface). To avoid overestimating the number of transitions to subterranean habitats, we further subdivided the interstitial and underground states based on the palaeovalleys in which species are restricted. A custom transition matrix was used to forbid dispersals of subterranean species among different palaeovalleys (see *Methods* and Supplementary Information, Supplementary Methods). Both reconstructions were simulated using ancestral character mapping with the r-package *phytools*^42^.

In the Australian clade ‘b” (Fig. 1), there were at least 42 transitions to the underground and two to the interstitial (clade “d”, see *Life underground*). These transitions were reconstructed as recent events, occurring within the last 5 Ma, with a few isolated shifts no older than 9 Ma.

The upper montane (>2800 m) to alpine taxa in New Guinea (clade “f”) derived from a common ancestor with a crown age of c. 10 Ma [95% HPD: 9.01–12.47], comprising at least 12 upper montane to alpine species, each endemic to different subregions of the central orogen. Notably, this region is under sampled, and additional discoveries can be expected. Alpine habitats are also present in New Zealand and Australia above c. 1400 m altitude, represented in our analysis by four species in clades “‘c” and “g” (*L. plicatus*, *L. gemellus*, *L. amabilis, L. praelargus*), all of which also inhabit lower elevations (Figs. 1 and 2).

Other New Guinea species originated since the late Miocene and are endemic to different regions of the lower montane band 1001–2000 m, except for one species restricted to tropical lowland forest (within clade “g”, 95% HPD: 1.29–2.09), subordinated in a clade of lower montane species.

We can sum up our findings in three distinct evolutionary trajectories for *Limbodessus*: (i) adaptation to living in the interstitial or below the surface (see *Life underground* below), (ii) adaptation to living in alpine habitats (see *Alpine habitats*), and iii) dispersal from west to east in the Pacific Ocean (see *Colonization of Oceania*).

### Life underground

Life beneath the Australian surface has witnessed significant evolutionary events since the late Miocene and especially Pliocene, as *Limbodessus* independently colonized groundwaters at least 42 times, as shown in Fig. 1, leading to at least 60 subterranean species. This colonization led to the potential for 18 speciation events within underground habitats, out of which 11 occurred within the same palaeovalleys (10 of which occurred in the same calcrete aquifers, see Fig. 1). Furthermore, we detected evidence of shared deleterious (i.e., frameshift and premature stop codons) mutations in vision-related genes in four pairs of sympatric sister species, highlighting that speciation occurred from an ancestral species already adapted to a subterranean groundwater lifestyle (i.e., stygobiotic ancestor) within isolated aquifers (Supplementary Information, Supplementary Results and Discussion and Table S3)^43^.

The climatic conditions during the early diversification of the genus were characterized by the middle Miocene climatic transition, shifting from the stable setting of the Oligocene/early Miocene to climatic cooling^44^ and aridification in Australia^41,45^. This climatic shift negatively impacted precipitation levels^46^, affecting hydroperiod dynamics and water availability^41^, and emerged as a key factor driving habitat specialization^47^. Consequently, faced with extremely arid environmental conditions and reduced surface water, species may have sought refuge in groundwaters as a survival strategy. This evolutionary scenario aligns with expectations under the Climatic Relict Hypothesis^37,48,49^, where following colonization by a pre-adapted surface species, cave populations become isolated due to climatic changes (e.g., glaciations or aridity) that cause the extinction of adjacent surface populations, promoting speciation of the cave population in allopatry. Indeed, the outstanding diversity of *Limbodessus* species within Australian groundwater has been previously related to this cause^37,50^, although our findings are based on a more robust phylogeny, using nuclear and mitochondrial markers, a dating approach using fossils, and a broader selection of surface taxa to investigate the transitions into the subterranean aquifers. Our results infer multiple parallel colonizations from epigean ancestors, but also provide strong support for “subterranean speciation”^43^, the latter potentially occurring in the presence of gene flow (Fig. 1 clade “b”, some of them also detected by Leijs et al.^50^). Within each aquifer, coexisting *Limbodessus* exhibit three distinct body sizes without overlap in accordance with the theory of limiting similarity^51^, facilitating assortative mating, which may have promoted speciation in the presence of gene flow.

Most groundwater taxa emerged during the last 4–8 Ma, just after the aridification process reached extremely arid conditions^41^, and was boosted after the brief return to wet conditions that reversed the long-term aridification trend c. 5 Ma^48^. These brief wet conditions may have caused *Limbodessus* to spread across Australia, with subsequent drying of surface aquatic habitats forcing species to migrate to subterranean habitats after water infiltration. This scenario is supported by the presence of a larger number of groundwater colonizations during the Pliocene and Pleistocene, although the emergence of several subterranean species was dated before the brief wet period. However, all of these apparent Late Miocene colonizations of subterranean habitats are represented by long terminal branches that may be an artifact of incomplete sampling (higher sampling could provide additional nodes leading to younger age estimates) or lineage extinction, which may be especially true for surface/interstitial taxa. Nonetheless, ancestral habitat reconstruction needs to be interpreted with caution because groundwater colonization is a random process^50^ that can occur anytime along isolated terminal branches. Furthermore, a single widespread surface species may have colonized different isolated groundwater islands, not necessarily at the same time, leading to multiple parallel speciation events. In fact, *Limbodessus* comprises related species from different palaeovalleys^52,53^ without groundwater connectivity between them, supporting the scenario of multiple colonizations from the surface.

The transition to life in groundwater ecosystems is associated with significant morphological changes (e.g., reduction of eyes and pigments, but gain of long tactile setae and antennae)^54,55^, and the reduction/loss of wings in subterranean beetles^56^. The precise origin of certain phenotypes associated with an underground lifestyle (i.e., reduction of eyes and pigments) remains insufficiently studied, leaving the question open as to whether these changes result from degeneration (or pseudogenization) of associated genes following relaxation of selective pressures^57^. Recent evidence suggests the neutral evolution of vision-related genes in diving beetles^58^, a phenomenon also observed in cavefish species^59^. Langille et al. demonstrated that modifications to vision-related genes (e.g., frameshift modifications or premature stop codons) have independently contributed to parallel vision degeneration in both *Limbodessus*^60^ and *Paroster*^61^. Another shared morphological feature of organisms adapted to live in aphotic habitats is a reduction in cuticle thickness and pigmentation (i.e., albinism). Although cuticle pigmentation is a selected trait involved in protection against ultraviolet light, crypsis, mimicry, or thermoregulation^62^, its selection may be relaxed in environments where species are not exposed to light, predation, or unstable environmental conditions^63^. In parallel with the convergence of eye reduction, groundwater species of *Limbodessus* exhibit convergence in both the reduction in cuticle thickness and cuticle pigmentation, which is practically transparent. Recent studies have demonstrated that this reduction in cuticle thickness serves as an enabling factor for cutaneous respiration in underground aquifers^64^.

The transition from the epigean ancestor to stygobiont (subterranean groundwater) specialists most likely occurred via adaptations to inhabit the interstitial environment of creek and river systems^65^. As seen previously by the expectation of the Climatic Relict Hypothesis, as epigean aquatic habitats diminished, aquatic beetles colonized first interstitial habitats before reaching groundwater. This transition has been documented for diving beetles^65,66^ where interstitial species show intermediate phenotypes, being less pigmented and with smaller eyes than epigean ones, but maintaining wings^37,50,65^. In *Limbodessus*, the known interstitial species are *L. occidentalis* from the desert/grassland climate zone in Western Australia and *L. rivulus* from tropical Queensland^66^. These are closely related (see in clade “d”) yet highly disjunct geographically (see distribution in Watts and Humphreys^67^). They are subordinated in a clade of stygobiont Western Australian species (Supplementary Information, Fig. S2). This hints at a widespread epigean ancestor of which we witness a fragmented, highly localized group of ecologically and morphologically derived species.

### Alpine habitats

The highest-elevation habitats across the distribution range of *Limbodessus* are limited to New Guinea, but alpine environments can also be found in Australia (including Tasmania) and New Zealand, from c. 1400 m altitude^68,69^. The latter habitats were colonized from 9 to 5 Ma by individual species (in clades “c” and “g”) that also occur in adjacent lower-altitude localities with temperate climates (Fig. 2). Notably, the mountain ranges and surrounding lowland regions of New Guinea predate these beetle lineages. This suggests a scenario where higher altitudes were colonized by cool-adapted local lowland populations.

In general, New Guinea features suitable stagnant water habitats in lowlands, large intramontane depressions, or valleys (e.g., the Western and Eastern Highland provinces) at altitudes around 1400–1900 m. The upper montane to alpine ecosystem of the New Guinea central orogen and several north coast mountains is vast, characterized by numerous geographically separated flat valleys with alpine meadows and peat swamps typically occurring from c. 3500 m to approximately 4000 m (also several areas with similar habitats at c. 2700 m, like Mount Hagen). Although specimens above 3900 m are absent, this may be due to undersampling or a lack of stagnant water above 3900 m in many regions. New Guinean species exhibit specific altitudinal preferences, with five being endemic to the 1000–2000 m montane band, and 12 are exclusively found above 2700 m. Also, the widespread *L. compactus* occurs from the lowland up to c. 1,400 m and has a broad predicted ecological niche model (Fig. 2), in particular compared to other species of the genus.

Most of the highest altitudes in New Guinea originated in the past c. 4 Ma. However, all the New Guinean alpine meadow endemics form clade “f” with a crown age of c. 10 Ma. Our analyses support the hypothesis of in situ diversification within this clade above 3000 m, linking all these species to alpine meadows. Ancestral area reconstruction and altitudinal preference analyses suggest that clade “f” initially split at 10 Ma into two lineages: a single species known from the Star Mountains, including the Mt. Juliana area, sister to a clade with the remaining 11 species. This latter group diversified only from c. 4 Ma along the central orogen. Geological evidence indicates that proto-Papuan archipelago, though not entirely flat, lacked developed alpine meadow systems or high valleys before c. 5 Ma^70^. Notably, none of the New Guinea lower-altitude montane species in clades “c” and “g” have relatives in the alpine meadows directly above the valleys they inhabit. Together, these findings support a model of in situ diversification at high elevations rather than colonization from low-altitude sources.

In our proposed scenario, we rule out the colonization of alpine meadows from a lowland source but rather suggest the passive uplift of two species during the central New Guinea orogeny from c. 8 Ma, followed by more recent, either range expansion/isolation or vicariance of ancestral species along the different mountain blocks. Also, the single lineage from Mt. Juliana lives sympatrically with a species from its sister clade, indicating a recent secondary colonization of the area. The evolution of alpine taxa by passive uplift is likely a common phenomenon, as suggested by Heads^69^, who argued that this is not easily statistically tested. For instance, some New Guinean *Taractrocera* butterflies^71^, as well as *Hapalopsittaca* parrots or monkey tree frogs in the Andes^72,73^, are believed to have undergone a similar speciation process associated with tectonic uplifting. Nevertheless, the present topology delivers evidence for this scenario.

Additionally, two of New Guinea’s lower-altitude montane species (the sister of clade “d” and the second species in clade “g”) originated c. 14–9 Ma. These might have also originated by passive uplift. Interestingly, none of these species are closely related to the high-altitude species occurring above their habitats, sometimes only a few kilometers away but at altitudes above 2700 m. The most recent three species of clade “g” contain one lowland species and two lower montane endemics; one of the montane species occurs in close geographic proximity with the lowland species. This might be a case of colonization from a lower altitude to the lower montane zone. Passive uplift (i.e., vicariance) might have to be ruled out in this case due to the recent origin of this clade c. 2 Ma, and speciation through climatic divergence is plausible^74,75^.

The ecomorphological transition in the high-altitude species led to melanism, with beetles being dark brown to black as opposed to the yellow to orange montane species, and the wings being reduced to short stubs^76^. The latter ecomorphological trait has proven to be a common feature in many high-elevation specialized taxa^77^ and is prevalent in the New Guinea beetle community adapted to alpine habitats^78^. As noted by Waters et al.^79^, abiotic stresses may cause dispersal reduction, resulting in isolation and, consequently, speciation. Many alpine meadow species also show strongly enlarged antennomeres in females^78^, otherwise only found in *L. curviplicatus* (Fiji, Samoa). However, its function remains unknown.

### Colonization of oceania

The colonization of Oceania was a random process, neither centered in a particular temporal nor topological dimension during cladogenesis. Despite this, the distribution of *Limbodessus,* primarily in the Sahul continent, revealed isolated colonization events in the Pacific archipelagos without a stepping-stone course. Fiji, for instance, was colonized c. 6 Ma by an Australian ancestor, leading to the evolution of *L. leveri*. Notably, this species evolved subsequent to colonization and isolation. This species is closely related to *L. compactus* (clade “c”), which concurrently underwent significant range expansion westward, reaching southern Japan. Thus, the stem lineage must have possessed significant dispersal capacity, perhaps reflected in the wide niche tolerance inferred for *L. compactus* (Fig. 2).

Moreover, Fiji and Samoa were also colonized by an ancestral species in New Guinea clade “g” around 5 Ma with subsequent colonization of French Polynesia. New Caledonia and Vanuatu were also colonized from New Guinea in c. 11 Ma. While the colonization of isolated archipelagos from New Guinea is remarkable, it does not necessitate extraordinary dispersal capabilities. Occasionally, reaching remote islands may occur through passive dispersal mechanisms such as wind currents^80^ or migratory birds^81^, serving as drivers of colonization.

## Conclusion

The diving beetles studied here demonstrated the ability to adapt to large-scale environmental changes in terms of aridification across Australia and the geotectonic origin of subalpine and alpine tropical habitats, mainly in New Guinea. In both cases, our findings suggest that these habitats, with their extreme environmental conditions, are most likely occupied by descendants of an in situ species pool rather than by dispersal from adjacent habitats. Research on the genomic background of this capacity to adapt to dramatic local change, as well as the potential to disperse and colonize over long stretches of the ocean, would be a valuable future research direction.

## Materials and methods

### Taxon sampling, DNA extraction, sequencing, and library processing

The comprehensive dataset of *Limbodessus* species stems from freshly sampled specimens as well as museum specimens from Zoologische Staatssammlung München (ZSM), the South Australia Museum (SAM), and the Western Australia Museum (WAM). In total, 57 samples representing one *Limbodessus* species each, some of them undescribed, were used, alongside four outgroups from related Bidessini genera (Supplementary Information, Table S2). Whole-genome sequencing libraries were prepared and sequenced on a NovaSeq 200 platform at StarSEQ (Mainz, Germany), targeting 25 paired-end million reads per sample, each with a read length of 150 base pairs. All raw data were uploaded to the NCBI public repository (Supplementary Information, Table S2). For each library, raw data underwent several processing steps, including quality control, adapter removal, genome assembly, and removal of contaminated contigs (Supplementary Information, Supplementary Methods). Assembled libraries were subsequently screened for UCEs following the *Phyluce* pipeline^82^ and the probe set designed by Gustafson et al.^83^ for the suborder Adephaga.

### Datasets

Two datasets were built for subsequent analyses. To establish a temporal framework of *Limbodessus* evolution and due to the absence of fossil records in this genus, we compiled an additional UCE dataset with the most comprehensive Dytiscidae data from NCBI (Supplementary Information, Table S1). This dataset contains 42 Dytiscidae species, featuring representatives of *Limbodessus* and related genera, along with one *Amphizoa* species as an outgroup. Raw data followed the same methodology as described above, considering a 50% completeness threshold within *Phyluce*.

A comprehensive dataset for *Limbodessus* was compiled by concatenating a selection of UCE loci (see below; Supplementary Information, Supplementary Methods) with the following commonly used molecular markers, including mitochondrial genomes and nuclear genes: rRNAs (5.8S, 18S, and 28S), histones (1, 2A, 2B, 3, and 4), arginine kinase, topoisomerase and wingless. Reference sequences of these markers were blasted^84^ against assembled libraries. Additional sequences were obtained from NCBI, including Sanger sequencing data and published transcriptomes^83,84^, plus newly generated data obtained by Sanger sequencing to soften missing data. This comprehensive dataset includes data of all described *Limbodessus* species but four (95% completeness).

### Phylogenetic analyses and calibration

To enhance our UCE datasets for phylogenetic analysis, we identified the 100 most clock-like UCEs (Supplementary Information, Supplementary Methods). This 100 UCEs dataset was analyzed to determine optimal partitions and evolutionary models for subsequent calibrated analyses.

Bayesian Inference analyses were conducted for the best partition scheme and evolutionary models, and a less computationally demanding evolutionary model (i.e., HKY + G + I), both under two alternative clock approaches, strict and uncorrelated lognormal priors. Our analyses were calibrated using the most comprehensive list of fossils available for Dytiscidae (Supplementary Information, Table S4). Analyzes were performed and compared using Path sampling/stepping-stone sampling scores^85,86^. The best analysis was chosen to extract three secondary calibration points for the following crown groups: *Limbodessus*, *Limbodessus* plus *Allodessus*, and *Bidessini*.

For the *Limbodessus* dataset, we performed additional model selection by considering two configurations: allowing all loci (UCEs plus commonly used molecular markers) to be pooled together in the same partitions (configuration 1) or using only the non-UCE loci (configuration 2). The best partition scheme and evolutionary models were used to reconstruct a calibrated Bayesian phylogenetic tree of *Limbodessus* using the secondary calibrations obtained in the Dytiscidae analyses under the two clock alternatives: strict and uncorrelated lognormal. The HKY + G + I model was also used as an alternative, less computationally intensive model. Path sampling/stepping-stone sampling scores were used for comparison and assessment of the analyses.

### Biogeographical analyzes

Ancestral area reconstruction analyses were performed in BioGeoBEARS^87^ for the *Limbodessus* dataset, considering five alternative hypotheses as detailed in Table 2: an unguided analysis (M0), two analyses with the standard hypothesis of New Guinean geological origin in the Miocene (M1 and M2) and two analyses with a relaxed origin of New Guinea in the Oligocene (M3 and M4). These hypotheses were tested using custom dispersal multiplier matrices that accounted for the proximity between the tested areas, ranging from 0.1 to 1, with a value of 0 for New Guinea before its emergence (i.e., standard hypothesis, Supplementary Information, Supplementary Methods, Table S5). Hypotheses M2 and M4 incorporated the use of the *w* parameter to soften the dispersal matrix values for hypotheses M1 and M3, respectively. Each hypothesis tested the DEC, DIVALIKE, and BAYAREALIKE models implemented in BioGeoBEARS, allowing for the founder-event speciation parameter *J* and a maximum of five areas per node. We used the maximum clade credibility tree from the selected Bayesian inference analysis, in combination with simplified distributions for the genus *Limbodessus*, which is widely distributed in Australia, Southeast Asia, and most of the Pacific archipelagos, with one species being also found in eastern Asia. In total, five areas were considered: East Asia (A), Australia (B), New Guinea (C), the Pacific archipelagos of New Caledonia, Vanuatu, Fiji, Samoa and French Polynesia (D), and New Zealand (E).

### Ancestral altitude potential and habitat reconstruction

Maximum altitudinal potential was tested using the discrete trait Markov model ARD (all rates different) in the *phytools* R-package^42^ using the UCEs plus other molecular markers phylogenetic trees. In total, five altitudinal ranges were considered, and species were assigned according to the maximum altitude in which they are found: below 1000 m; 1001–2000 m; 2001–3000 m; 3001–4000 m; and >4001 m. These classifications are inherently subjective to some degree. We focused particularly on the New Guinea landmass, which possess the highest elevations, the most species diversity at high elevations, and the most extensive montane habitat in the region. The rationale for the altitudinal categorization was as follows: below 1,000 m, stagnant water habitats suitable for these beetles are confined to lowland swamps, inundation forest, and riverbank puddles; the range from 1001 to 2000 m includes higher intramontane depressions or valley complexes featuring lake-associated and riverine wetlands; the 2001 to 3000 m range generally offers fewer suitable habitats, some depressions close to 3000 m feature small peat swamps and inundated forest; the 3001–4000 m band includes extensive stretches of alpine meadows and peat swamps; finally, habitat above 4001 m are limited and poorly explored, characterized by rocky or gravelly pools in the alpine zone.

Habitat was also reconstructed considering whether species are found in subterranean, interstitial or epigean habitats. The colonization of groundwater has previously been studied^37,50^, leading to a likely independent transition and very limited intra-calcrete speciation (i.e., potential sympatric or parapatric speciation, although limited micro-allopatry within the calcrete could still take place due to fluctuations of the water table). Although the calcretes in which species live are isolated, their spatial connectivity may have been different in the past, and thus, our model allows dispersal among calcretes in the same palaeovalleys (Supplementary Information, Supplementary Methods). Additional states were included to consider epigean and interstitial species. The custom model considers that transitions from interstitial to subterranean habitats are only possible within the same palaeovalley as previously suggested^61^.

In both cases, ancestral character mapping was then simulated using the *make.simmap* function in the r-package *phytools*^42^, considering 1000 simulations using the consensus topology and forcing the root state to be an epigean species below 2000 m due to the unavailability of higher elevations in the area during the emergence of *Limbodessus* and the unlikely subterranean origin. The adaptations in *Limbodessus* are highly specialized for subterranean environments, including extreme morphological changes such as loss of pigmentation, reduction or absence of vision^52^ and wings^53^, and specialized metabolic processes^64,88^ (see *Life underground*), making reversal to epigean habitat very unlikely, as these traits are typically irreversible and maladaptive in surface environments.

### Evolutionary trajectory of phototransduction genes

Blast^84^ was used to identify seven phototransduction genes from the newly sequenced whole-genome sequencing libraries plus data from genomic analyses of these genes by Langille et al.^60^. Exon sequences were aligned and translated into amino acid sequences using Geneious v10.2.6^89^, and alignments were visualized to detect shared deleterious mutations (i.e., frameshift mutations and premature stop codons) in the phototransduction genes of sympatric sister species (Supplementary Information, Table S3).

### Ecological niche modeling

We used presence-only data collected by the authors to model ecological niches and highlight the present-day potential distribution of some *Limbodessus* species (Supplementary Information, Supplementary Methods).

### Statistics and reproducibility

All analyses were conducted as described in each Methods subsection, with further specifications provided in the Supplementary Methods. Phylogenetic analyses were performed using a combination of UCEs, mitochondrial genomes, and 11 nuclear genes for 95 different species. The input alignment used for these analyses is provided in the Open Science Framework repository (see “Data availability”). Biogeographical analyses employed the BioGeoBears package in R^87,90^, evaluating alternative biogeographical models. Ancestral altitude potential and habitat reconstructions were performed in R^90^ using the *phytools* package^23^ based on the consensus topology, with 1000 simulation replicates. Detailed methods, along with the consensus topology (available in the Open Science Framework repository, see Data availability), ensure full reproducibility of the analyses.

### Reporting summary

Further information on research design is available in the Nature Portfolio Reporting Summary linked to this article.

## Supplementary information

Supplementary information file revised

Reporting summary

## Data Availability

DNA sequences generated during this study are uploaded to the NCBI database. Accession numbers are available in the Supplementary Information. Alignment data, vision-related gene sequences, and biogeographical results are deposited in the Open Science Framework: 10.17605/OSF.IO/NJXRM.

## References

[1] de Carbonnières, L. R. *Observations faites dans les Pyrénées, pour servir de suite à des observations sur les Alpes, insérées dans une traduction des Lettres de W Coxe*, *sur la Suisse*. (Belin, 1789).

[2] Humboldt, A. “von.” *Voyage de Humboldt et Bonpland*: Essai Sur La Géographie Des Plantes, Accompagné D’Un Tableau Physique Des Régions Équinoxiales : Fondé sur des mesures exécutées, depuis le dixième degré de latitude boréale jusqu’au dixième degré de latitude australe, *pendant les* années 1799, *1800*, 1801, *1802* et *1803* / Par Al. De Humboldt Et A. Bonpland; Rédigé Par Alexandre De Humboldt. (Schoell, 1807).

[3] Moret, P., Muriel, P., Jaramillo, R. & Dangles, O. Humboldt’s Tableau Physique revisited. *Proc. Natl Acad. Sci. USA* **116**, 12889–12894 (2019). 31138688 10.1073/pnas.1904585116 PMC6601271

[4] Southwood, T. R. E. Habitat, the templet for ecological strategies? *J. Anim. Ecol.* **46**, 337–365 (1977).

[5] Southwood, T. R. E. Tactics, strategies and templets. *Oikos* **52**, 3–18 (1988).

[6] Condamine, F. L., Rolland, J. & Morlon, H. Macroevolutionary perspectives to environmental change. *Ecol. Lett.* **16**, 72–85 (2013). 23331627 10.1111/ele.12062

[7] Simpson, G. G. *The Major Features of Evolution*. (Columbia University Press, 1953).

[8] Losos, J. B., Warheitt, K. I. & Schoener, T. W. Adaptive differentiation following experimental island colonization in *Anolis* lizards. *Nature* **387**, 70–73 (1997).

[9] Boschman, L. M. & Condamine, F. L. Mountain radiations are not only rapid and recent: Ancient diversification of South American frog and lizard families related to Paleogene Andean orogeny and Cenozoic climate variations. *Glob. Planet Change* **208**, 103704 (2022).

[10] Badgley, C. et al. Biodiversity and topographic complexity: modern and geohistorical perspectives. *Trends Ecol. Evol.* **32**, 211–226 (2017). 28196688 10.1016/j.tree.2016.12.010 PMC5895180

[11] Perrigo, A., Hoorn, C. & Antonelli, A. Why mountains matter for biodiversity. *J. Biogeogr.* **47**, 315–325 (2020).

[12] Heads, M. Passive uplift of plant and animal populations during mountain-building. *Cladistics* **35**, 550–572 (2019). 34618940 10.1111/cla.12368

[13] Lohman, D. J. et al. Biogeography of the Indo-Australian archipelago. *Annu. Rev. Ecol. Evol. Syst.* **42**, 205–226 (2011).

[14] Toussaint, E. F. A. et al. New Guinean orogenic dynamics and biota evolution revealed using a custom geospatial analysis pipeline. *BMC Ecol. Evol.* **21**, 51 (2021). 33823805 10.1186/s12862-021-01764-2 PMC8022562

[15] Neall, V. E. & Trewick, S. A. The age and origin of the Pacific islands: a geological overview. *Philos. Trans. R. Soc. Lond. B Biol. Sci.* **363**, 3293–3308 (2008). 18768382 10.1098/rstb.2008.0119 PMC2607379

[16] Roycroft, E. et al. New Guinea uplift opens ecological opportunity across a continent. *Curr. Biol.* **32**, 4215–4224.e3 (2022). 36057260 10.1016/j.cub.2022.08.021

[17] Lambers, H. *On the Ecology of Australia’s Arid Zone*. 388 (Springer, 2018).

[18] Baldwin, S. L., Fitzgerald, P. G. & Webb, L. E. Tectonics of the New Guinea region. *Annu. Rev. Earth Planet Sci.* **40**, 495–520 (2012).

[19] Cloos, M. et al. *Collisional Delamination in New Guinea: the Geotectonics of Subducting Slab Breakoff*. 400. (Geological Society of America, 2005).

[20] Toussaint, E. F. A. et al. The towering orogeny of New Guinea as a trigger for arthropod megadiversity. *Nat. Commun*. **5**, 4001 (2014). 10.1038/ncomms5001 24874774

[21] Toussaint, E. F. A., Sagata, K., Surbakti, S., Hendrich, L. & Balke, M. Australasian sky islands act as a diversity pump facilitating peripheral speciation and complex reversal from narrow endemic to widespread ecological supertramp. *Ecol. Evol.* **3**, 1031–1049 (2013). 23610642 10.1002/ece3.517 PMC3631412

[22] Cámara-Leret, R. et al. New Guinea has the world’s richest island flora. *Nature* **584**, 579–583 (2020). 32760001 10.1038/s41586-020-2549-5

[23] Gold, D. P., White, L. T., Gunawan, I. & BouDagher-Fadel, M. K. Relative sea-level change in western New Guinea recorded by regional biostratigraphic data. *Mar. Pet. Geol.* **86**, 1133–1158 (2017).

[24] Letsch, H. et al. Beetle evolution illuminates the geological history of the World’s most diverse tropical archipelago. *Ecography (Cop.)* **2023**, e06898 (2023).

[25] Harrington, L et al. Tectonic, geodynamic and surface process driving forces of Australia’s paleogeography since the Jurassic. in *Petroleum Exploration Society of Australia Symposium* (2019).

[26] Sniderman, J. M. K. et al. Pliocene reversal of late Neogene aridification. *Proc. Natl Acad. Sci. USA* **113**, 1999–2004 (2016). 26858429 10.1073/pnas.1520188113 PMC4776468

[27] Humphreys, W. F. Rising from Down Under: developments in subterranean biodiversity in Australia from a groundwater fauna perspective. *Invertebr. Syst.* **22**, 85 (2008).

[28] Mani, M. S. Other Tropical Mountains. in *Ecology and Biogeography of High Altitude Insects* (ed Mani, M. S.) 176–195 (Springer Netherlands, Dordrecht, 1968).

[29] Deiner, K., Lemmon, A. R., Mack, A. L., Fleischer, R. C. & Dumbacher, J. P. A Passerine Bird’s evolution corroborates the geologic history of the island of New Guinea. *PLoS ONE* **6**, e19479 (2011). 21573115 10.1371/journal.pone.0019479 PMC3089620

[30] Eldridge, M. D. B. et al. Phylogenetic analysis of the tree-kangaroos (*Dendrolagus*) reveals multiple divergent lineages within New Guinea. *Mol. Phylogenet. Evol.* **127**, 589–599 (2018). 29807156 10.1016/j.ympev.2018.05.030

[31] Meredith, R. W., Mendoza, M. A., Roberts, K. K., Westerman, M. & Springer, M. S. A phylogeny and timescale for the evolution of Pseudocheiridae (Marsupialia: Diprotodontia) in Australia and New Guinea. *J. Mamm. Evol.* **17**, 75–99 (2010). 21125022 10.1007/s10914-010-9129-7 PMC2987229

[32] Unmack, P. J., Allen, G. R. & Johnson, J. B. Phylogeny and biogeography of rainbowfishes (Melanotaeniidae) from Australia and New Guinea. *Mol. Phylogenet. Evol.* **67**, 15–27 (2013). 23313459 10.1016/j.ympev.2012.12.019

[33] Surbakti, S., Balke, M. & Hendrich, L. *Limbodessus moni* sp. nov., a new high altitudinal diving beetle from the Grasberg in West Papua, Indonesia (Coleoptera: Dytiscidae, Bidessini). *Zootaxa* **5319**, 413–420 (2023). 37518222 10.11646/zootaxa.5319.3.7

[34] Austin, A. et al. The unique Australian subterranean dytiscidae: diversity, biology, and evolution. in *Ecology, Systematics, and the Natural History of Predaceous Diving Beetles (Coleoptera: Dytiscidae)* (ed Yee, D. A.) 401–426 (Springer, 2023).

[35] Balke, M., Watts, C. H. S., Cooper, S. J. B., Humphreys, W. F. & Vogler, A. P. A highly modified stygobiont diving beetle of the genus *Copelatus* (Coleoptera, Dytiscidae): taxonomy and cladistic analysis based on mitochondrial DNA sequences. *Syst. Entomol.* **29**, 59–67 (2004).

[36] Peck, S. B. & Finston, T. L. Galapagos islands troglobites: the questions of tropical troglobites, parapatric distributions with eyed-sister-species, and their origin by parapatric speciation. *Mem. Biospeol.* **20**, 19–37 (1993).

[37] Leys, R., Watts, C. H. S., Cooper, S. J. B. & Humphreys, W. F. Evolution of subterranean diving beetles (Coleoptera: Dytiscidae: Hydroporini, Bidessini) in the arid zone of Australia. *Evolution* **57**, 2819–2834 (2003). 14761060 10.1111/j.0014-3820.2003.tb01523.x

[38] Balke, M. & Ribera, I. Jumping across Wallace’s line: *Allodessus* Guignot and *Limbodessus* Guignot revisited (Coleoptera: Dytiscidae, Bidessini) based on molecular-phylogenetic and morphological data. *Aust. J. Entomol.* **43**, 114–128 (2004).

[39] Frakes, L. A., McGowran, B. & Bowler, J. M. Evolution of Australian Environments. in *Fauna of Australia. General Articles* (eds Dyne, G. R. & Walton, D. W.) 1–17 (Australian Government Publishing Service, 1987).

[40] Müller, R. et al. Formation of Australian continental margin highlands driven by plate–mantle interaction. *Earth Planet Sci. Lett.* **441**, 60–70 (2016).

[41] Byrne, M. et al. Birth of a biome: insights into the assembly and maintenance of the Australian arid zone biota. *Mol. Ecol.* **17**, 4398–4417 (2008). 18761619 10.1111/j.1365-294X.2008.03899.x

[42] Revell, L. J. phytools: An R package for phylogenetic comparative biology (and other things). *Methods Ecol. Evol.* **3**, 217–223 (2012).

[43] Cooper, S. J. B. et al. Phylogenies reveal speciation dynamics: case studies from groundwater. in *Groundwater Ecology and Evolution* (eds Malard, F., Griebler, C. & Retaux, S.) 165–183 (Elsevier, 2023).

[44] Flower, B. P. & Kennett, J. P. The middle Miocene climatic transition: East Antarctic ice sheet development, deep ocean circulation and global carbon cycling. *Palaeogeogr. Palaeoclimatol. Palaeoecol.* **108**, 537–555 (1994).

[45] Martin, H. A. Cenozoic climatic change and the development of the arid vegetation in Australia. *J. Arid Environ.* **66**, 533–563 (2006).

[46] Retallack, G. J. Cenozoic paleoclimate on land in North America. *J. Geol.* **115**, 271–294 (2007).

[47] Villastrigo, A., Fery, H., Manuel, M., Millán, A. & Ribera, I. Evolution of salinity tolerance in the diving beetle tribe Hygrotini (Coleoptera, Dytiscidae). *Zool. Scr.* **47**, 63–71 (2018).

[48] Barr, T. C., Jr. Cave ecology and the evolution of troglobites. in *Evolutionary Biology* 35–102 (Springer US, Boston, MA, 1968).

[49] Barr, T. C. Jr & H olsinger, J. R. Speciation in cave faunas. *Annu. Rev. Ecol. Syst.* **16**, 313–337 (1985).

[50] Leijs, R. et al. Evolution of blind beetles in isolated aquifers: a test of alternative modes of speciation. *PLoS ONE* **7**, 34260 (2012). 10.1371/journal.pone.0034260 PMC3316697 22479581

[51] Vergnon, R., Leijs, R., van Nes, E. H. & Scheffer, M. Repeated parallel evolution reveals limiting similarity in subterranean diving beetles. *Am. Nat.* **182**, 67–75 (2013). 23778227 10.1086/670589

[52] Cooper, S. J. B., Hinze, S., Leys, R., Watts, C. H. S. & Humphreys, W. F. Islands under the desert: molecular systematics and evolutionary origins of stygobiontic water beetles (Coleoptera: Dytiscidae) from central Western Australia. *Invertebr. Syst.* **16**, 589–598 (2002).

[53] Watts, C. H. S. & Humphreys, W. F. Twenty-six new Dytiscidae (Coleoptera) of the genera *Limbodessus* Guignot and *Nirripirti* Watts & Humphreys, from underground waters in Australia. *Trans. R. Soc. S. Aust.* **130**, 123–185 (2006).

[54] Culver, D. C. & Pipan, T. *The Biology of Caves and Other Subterranean Habitats the Biology of Caves and Other Subterranean Habitats*. (Oxford University Press, London, England, 2019).

[55] Zhao, Y. et al. Evolutionary transition from surface to subterranean living in Australian water beetles (Coleoptera, Dytiscidae) through adaptive and relaxed selection. *Biol. J. Linn. Soc.* **142**, 280–293 (2024).

[56] Miller, K. B. & Bergsten, J. *Diving Beetles of the World: Systematics and Biology of the Dytiscidae*. 320 (Johns Hopkins University Press, Baltimore, 2016).

[57] Zhang, J. Neutral theory and phenotypic evolution. *Mol. Biol. Evol.* **35**, 1327–1331 (2018). 29659993 10.1093/molbev/msy065 PMC5967557

[58] Cooper, S. et al. Phylogenies reveal speciation dynamics: case studies from groundwater. in *Groundwater Ecology and Evolution**(**Second Edition**)* (eds Malard, F., Griebler, C. & Rétaux, S.) 165–183 (Academic Press, San Diego, 2023).

[59] Niemiller, M. L., Fitzpatrick, B. M., Shah, P., Schmitz, L. & Near, T. J. Evidence for repeated loss of selective constraint in rhodopsin of amblyopsid cavefishes (Teleostei: Amblyopsidae). *Evolution* **67**, 732–748 (2013). 23461324 10.1111/j.1558-5646.2012.01822.x

[60] Langille, B. L. et al. Parallel decay of vision genes in subterranean water beetles. *Mol. Phylogenet. Evol.* **173**, 107522 (2022). 35595008 10.1016/j.ympev.2022.107522

[61] Langille, B. L. et al. Evidence for speciation underground in diving beetles (Dytiscidae) from a subterranean archipelago. *Evolution* **75**, 166–175 (2021). 33219700 10.1111/evo.14135

[62] True, J. R. Insect melanism: the molecules matter. *Trends Ecol. Evol.* **18**, 640–647 (2003).

[63] Bilandžija, H., Ma, L., Parkhurst, A. & Jeffery, W. R. A potential benefit of albinism in *Astyanax* cavefish: downregulation of the *oca2* gene increases tyrosine and catecholamine levels as an alternative to melanin synthesis. *PLoS ONE* **8**, e80823 (2013). 24282555 10.1371/journal.pone.0080823 PMC3840000

[64] Jones, K. K., Cooper, S. J. B. & Seymour, R. S. Cutaneous respiration by diving beetles from underground aquifers of Western Australia (Coleoptera: Dytiscidae). *J. Exp. Biol.* **222**, 1–13 (2019). 10.1242/jeb.196659 30948497

[65] Watts, C. H. S., Hendrich, L. & Balke, M. A new interstitial species of diving beetle from tropical northern Australia provides a scenario for the transition of epigean to stygobitic life (Coleoptera, Dytiscidae, Copelatinae). *Subterr. Biol.* **19**, 23–29 (2016).

[66] Larson, D. J. *Boongurrus rivulus*, a new genus and species of water beetle (Coleoptera: Dytiscidae: Bidessini) from northern Queensland, Australia. *Aust. J. Entomol.* **33**, 217–221 (1994).

[67] Watts, C. H. S. & Humphreys, W. F. Fourteen new dytiscidae (Coleoptera) of the genera *Limbodessus* Guignot, Paroster Sharp, and *Exocelina* Broun from underground waters in Australia. *Trans. R. Soc. S. Aust.* **133**, 62–107 (2009).

[68] Buckley, T. R., Hoare, R. J. B. & Leschen, R. A. B. Key questions on the evolution and biogeography of New Zealand alpine insects. *J. R. Soc. N. Z.* **54**, 30–54 (2024). 39439474 10.1080/03036758.2022.2130367 PMC11459838

[69] Williams, R. J. et al. Alpine ecosystems. in *Biodiversity and Environmental Change: Monitoring, Challenges and Directions* (eds Lindenmayer, D., Burns, E., Thurgate, N. & Lowe, A.) 167–212 (CSIRO Publishing Melbourne, 2014).

[70] van Ufford, A. Q. & Cloos, M. Cenozoic tectonics of New Guinea. *AAPG Bull.* **89**, 119–140 (2005).

[71] De, J. R. Phylogeny and biogeography of the genus *Taractrocera* Butler, 1870 (Lepidoptera: Hesperiidae), an example of Southeast Asian-Australian interchange. *Zool. Meded.* **78**, 383–415 (2004).

[72] Quintero, E., Ribas, C. C. & Cracraft, J. The Andean *Hapalopsittaca* parrots (Psittacidae, Aves): an example of montane‐tropical lowland vicariance: Biogeography of *Hapalopsittaca* parrots. *Zool. Scr.* **42**, 28–43 (2013).

[73] Almeida-Silva, D., Servino, L. M., Pontes-Nogueira, M. & Sawaya, R. J. Marine introgressions and Andean uplift have driven diversification in neotropical Monkey tree frogs (Anura, Phyllomedusinae). *PeerJ* **12**, e17232 (2024). 38646479 10.7717/peerj.17232 PMC11027904

[74] Cadena, C. D. et al. Latitude, elevational climatic zonation and speciation in New World vertebrates. *Proc. Biol. Sci.* **279**, 194–201 (2012). 21632626 10.1098/rspb.2011.0720 PMC3223651

[75] Kozak, K. H. & Wiens, J. J. Climatic zonation drives latitudinal variation in speciation mechanisms. *Proc. Biol. Sci.* **274**, 2995–3003 (2007). 17895224 10.1098/rspb.2007.1106 PMC2291165

[76] Balke, M. et al. Two new species of Limbodessus diving beetles from New Guinea - short verbal descriptions flanked by online content (digital photography, μCT scans, drawings and DNA sequence data). *Biodivers. Data J.* **3**, e7096 (2015). 10.3897/BDJ.3.e7096 PMC4700388 26752969

[77] Roff, D. A. The evolution of flightlessness in insects. *Ecol. Monogr.* **60**, 389–421 (1990).

[78] Darlington, P. J. The carabid beetles of New Guinea. Part IV. General considerations; analysis and history of fauna; taxonomic supplement. *Bull. Mus. Comp. Zool.* **142**, 129–339 (1971).

[79] Waters, J. M., Emerson, B. C., Arribas, P. & McCulloch, G. A. Dispersal reduction: causes, genomic mechanisms, and evolutionary consequences. *Trends Ecol. Evol.* **35**, 512–522 (2020). 32396818 10.1016/j.tree.2020.01.012

[80] Gillespie, R. G. et al. Long-distance dispersal: a framework for hypothesis testing. *Trends Ecol. Evol.* **27**, 47–56 (2012). 22014977 10.1016/j.tree.2011.08.009

[81] Fery, H. & Challet, G. *Hygrotus* (*Coelambus*) *nubilus* (LeConte, 1855) on Mauna Kea (Hawaii) – first record of the genus from the Pacific zoogeographical region (Coleoptera: Dytiscidae). *Linz Biol. Beitr.* **47**, 1303–1309 (2015).

[82] Faircloth, B. C. PHYLUCE is a software package for the analysis of conserved genomic loci. *Bioinformatics* **32**, 786–788 (2016). 26530724 10.1093/bioinformatics/btv646

[83] Gustafson, G. T. et al. Ultraconserved element (UCE) probe set design: Base genome and initial design parameters critical for optimization. *Ecol. Evol.* **9**, 6933–6948 (2019). 31312430 10.1002/ece3.5260 PMC6617817

[84] Madden, T. L. et al. BLAST+: architecture and applications. *BMC Bioinforma.* **10**, 421 (2009). 10.1186/1471-2105-10-421 PMC2803857 20003500

[85] Baele, G. et al. Improving the accuracy of demographic and molecular clock model comparison while accommodating phylogenetic uncertainty. *Mol. Biol. Evol.* **29**, 2157–2167 (2012). 22403239 10.1093/molbev/mss084 PMC3424409

[86] Baele, G. & Lemey, P. Bayesian evolutionary model testing in the phylogenomics era: matching model complexity with computational efficiency. *Bioinformatics* **29**, 1970–1979 (2013). 23766415 10.1093/bioinformatics/btt340

[87] Matzke, N. J. *Probabilistic Historical Biogeography: New Models for Founder-Event Speciation, Imperfect Detection, and Fossils Allow Improved Accuracy and Model-Testing*. (University of California, 2013).

[88] Jones, K. K. et al. The critical thermal maximum of diving beetles (Coleoptera: Dytiscidae): a comparison of subterranean and surface-dwelling species. *Curr. Res. Insect Sci.* **1**, 100019 (2021). 36003597 10.1016/j.cris.2021.100019 PMC9387432

[89] Kearse, M. et al. Geneious basic: an integrated and extendable desktop software platform for the organization and analysis of sequence data. *Bioinformatics* **28**, 1647–1649 (2012). 22543367 10.1093/bioinformatics/bts199 PMC3371832

[90] R Core Team. *R: A Language and Environment for Statistical Computing*. https://www.R-project.org/ (2023).

