# Supplementary material for: Aridification and major geotectonic landscape change shaped an extraordinary species radiation across a world’s extreme elevational gradient
Source: Commun Biol. 2024 Nov 13;7:1500. doi: 10.1038/s42003-024-07181-7 (PMC11561355; doi:10.1038/s42003-024-07181-7)
Supplement: Supplementary file 1 — Supplementary information file revised [file 42003_2024_7181_MOESM1_ESM.pdf]

Supplementary Information for

Aridification and major geotectonic landscape change shaped an extraordinary species radiation across a world's extreme elevational gradient

Adrián Villastrigo[1*], Steven J.B. Cooper[2,3], Barbara Langille[3], Erinn P. Fagan-Jeffries[3], William F. Humphreys[4,5,], Lars Hendrich[1], Michael Balke[1,6]

[1]Division of Entomology, SNSB-Zoologische Staatssammlung München, Munich, Germany
[2]South Australia Museum, Adelaide, South Australia, Australia
[3]Department of Ecology and Evolutionary Biology, School of Biological Sciences, and Environment Institute, The University of Adelaide, Adelaide, South Australia, Australia
[4]Western Australian Museum, Welshpool DC, Western Australia, Australia
[5]School of Biological Sciences, University of Western Australia, Crawley, Western Australia, Australia
[6]GeoBioCenter, Ludwig Maximilians University, Munich, Germany

*Corresponding author: adrianvillastrigo@um.es

This file includes:

Supplementary Methods

Supplementary Results and Discussion

Supplementary Figures S1 to S7

Supplementary Tables S1 to S8

References

Supplementary Methods

Taxon sampling and DNA extraction

Fresh and museum specimens were obtained from the Zoologische Staatssammlung München (ZSM), the South Australia Museum (SAM), and the Western Australia Museum (WAM) (Supplementary Information, Table S2). DNA was extracted following a non-destructive protocol using a NucleoSpin Tissue Kit (Macherey-Nagel) as outlined in Villastrigo *et al*.[1]. A total of 57 *Limbodessus* species, some of them undescribed, were used, along with the addition of four outgroups from the related Bidessini genera *Papuadessus*, *Liodessus* and *Allodessus* (Supplementary Information, Table S2). DNA extracts were quantified using QuBit High-Sensitivity 2.0, followed by library construction using the NEBNext Ultra II FS DNA library kit. Representatives from the Moluccas (*Limbodessus skalei*) and the Sulawesi Island did not yield sufficient amounts of data for this study.

Sequencing, assembling, decontamination and ultraconserved elements (UCE) extraction

DNA templates were used to generate low-coverage whole-genome sequences. Libraries were sequenced on a NovaSeq 200 platform at StarSEQ (Mainz, Germany) targeting 25 paired-end million reads per sample and a read length of 150 base pairs. All raw data were deposited in the NCBI public repository (Supplementary Information, Table S2). For each library, raw data quality was assessed using FastQC (https://www.bioinformatics.babraham.ac.uk/projects/fastqc/). Adapters were removed and reads with poor quality values and low length were trimmed using BBDuk from the BBTools suite (https://jgi.doe.gov/data-and-tools/software-tools/bbtools/). As some libraries were generated from very low input DNA (e.g., *Limbodessus* spD, below the detection threshold of High Sensitivity QuBit), we masked reads (entropy below 0.01), which addressed poly-G artifacts. Libraries were assembled *de novo* using SPAdes v3.15.3[2] and decontaminated using Blobtools v1.1.1[3]. Contigs were checked against the NCBI *nt* database (e-value cutoff of 1e-25). Putative contaminations comprised Proteobacteria, Actinobacteria, and chordates, reaching up to 2.1% of the total contigs (most of the contaminants belonged to samples from the early 1990s, with a mean contamination of 0.57% when those samples were excluded; results not shown).

Assembled libraries were checked for UCEs following the *Phyluce* pipeline[4] and the probe set designed by Gustafson *et al*.[5] for the suborder Adephaga. We considered distinct completeness thresholds for our dataset: 50, 60, and 70 per cent completeness for topological examination under IQTree v.2.1.3[6].

Datasets

Two datasets were built for subsequent analyses. Due to the absence of fossil record in the study group, we compiled an additional UCE dataset with the most comprehensive Dytiscidae data from NCBI, mainly from Baca *et al*.[7] and Vasilikopoulos *et al*.[8]. This dataset (Supplementary Information, Table S1) contains 42 Dytiscidae species (including five of our newly sequenced samples representing both *Limbodessus* and related genera within the same tribe) and one *Amphizoa* species as an outgroup. Raw data were retrieved, and the same methodology as described above was followed. Due to the different data sources for this dataset, we considered a 50 % completeness threshold within *Phyluce*.

A comprehensive dataset was compiled for *Limbodessus* by concatenating the 100 most clock-likeliness UCE loci (see below) plus commonly used molecular markers, including mitochondrial genomes and nuclear genes: rRNAs (5.8S, 18S and 28S), histones (1, 2A, 2B, 3 and 4), arginine kinase, topoisomerase and wingless. Reference sequences of these markers were blasted[9] against assembled libraries. Additional sequences were obtained from NCBI, including Sanger sequencing data and published transcriptomes[10]. Additional sequences were generated using Sanger sequencing for the histone 3, arginine kinase, topoisomerase and wingless genes. Furthermore, COI sequences were targeted to integrate missing species into the dataset, as an effective methodology to incorporate missing taxa without genomic data[11]. This comprehensive dataset includes data of all described *Limbodessus* species but four (95% completeness).

Phylogenetic analyses and calibration

To enhance the UCE datasets for phylogenetic analyzes avoiding misleading loci and, thus, potential systematic biases[12], we selected the 100 most clock-like UCEs. For that, we used genesortR[13] in the Dytiscidae dataset, sorting UCEs based on 12 properties, four of which focused on increasing the clock-

likeliness of our data: root-to-tip variance, average patristic distances, level of saturation, and compositional heterogeneity. We used IQTree v2.1.3[6] and its model selection approach[14] to obtain individual UCE trees, as required by genesortR (Supplementary Information, Fig. S1). This 100 UCEs dataset was analyzed in PartitionFinder2[15] to select the best partitions and evolutionary models in the calibration analyzes. To avoid overparameterization and reduce computational time, we included a minimum partition size of 2,000 nucleotides ("rcluster" approach under the "min-subset-size" parameter).

First, BEAST v1.10.4[16] was run for the best partition scheme and evolutionary models, including a less computationally demanding evolutionary model (i.e., HKY+G+I) and one molecular clock per partition, under two alternative approaches: strict and uncorrelated lognormal priors. Our analyzes were calibrated using the most comprehensive list of fossils available for Dytiscidae, recently compiled from a broader analysis[7] and widely accepted to the water beetles' specialist criteria [17]. In total, eight fossils within Dytiscidae were selected (Supplementary Information, Table S4) and analyzed following lognormal and exponential priors. In total, eight analyzes were performed and compared using Path sampling / Stepping-stone sampling scores[18,19]. The best analysis was used to extract three secondary calibration points for the following crown groups: *Limbodessus*, *Limbodessus* plus *Allodessus*, and Bidessini.

For the *Limbodessus* dataset, we performed additional PartitionFinder2 analyzes using the same configuration but allowing all loci (UCEs plus commonly used molecular markers) to be pooled together in the same partitions or using only the non-UCE loci. The best partition scheme and evolutionary models based on the PartitionFinder2 analyzes (configuration 1: all loci pooled together, configuration 2: UCE and non-UCE loci concatenated but analyzed separately) were used to reconstruct the phylogenetic tree in BEAST of *Limbodessus* using the four secondary calibrations obtained in the Dytiscidae analyzes. We considered under two clock priors: strict and uncorrelated lognormal. Again, the evolutionary model HKY+G+I was used as an alternative less computationally intensive model. Analyzes were then compared using Path sampling / Stepping-stone sampling scores.

Biogeographical analyzes

Ancestral area reconstruction analyzes were performed in BioGeoBears[20] for the *Limbodessus* dataset, considering five alternative hypotheses (Table 2): an unguided analysis (M0), two analyses with the standard hypothesis of New Guinean geological origin in the Miocene[21] (M1 and M2) and two analyses with a relaxed origin of New Guinea in the Oligocene[22] (M3 and M4). These hypotheses were tested using custom dispersal multiplier matrices that accounted for the proximity between the tested areas, with values ranging from 0.1 to 1. A dispersal rate of 1 was applied to regions that were contiguous and highly connected (e.g., the Sahul and East Asia, which were separated by less than 100 km and share the widespread species *Limbodessus compactus*). A value of 0.5 was assigned to moderately connected regions but not contiguous (e.g., Australia and New Zealand in present-day configurations). Finally, a dispersal rate of 0.1 was used for highly disconnected and non-contiguous areas (e.g., New Guinea and New Zealand in the present time). Prior to its emergence, New Guinea was assigned a value of 0 (i.e., standard hypothesis, Supplementary Information, Table S5). Hypotheses M2 and M4 incorporated the use of the *w* parameter to soften the dispersal multiplier matrices for hypotheses M1 and M3, respectively. Each hypothesis tested the DEC, DIVALIKE and BAYAREALIKE models implemented in BioGeoBEARS, allowing for the founder-event speciation parameter *J* and a maximum of five areas per node. Despite the current limitations and uncertainty regarding the validity of the founder-event speciation calculation[23], we compared the best model with and without the *J* parameter based on AIC scores. We used the maximum clade credibility tree from the selected Bayesian inference analysis, in combination with simplified distributions for the genus *Limbodessus*, which is widely distributed in Australia, Southeast Asia, and most of the Pacific archipelagos, with one species being also found in eastern Asia. In total, five areas were considered: East Asia (A), Australia (B), New Guinea (C), the Pacific archipelagos of New Caledonia, Vanuatu, Fiji, Samoa and French Polynesia (D), and New Zealand (E).

Ancestral altitude potential and habitat reconstruction

Maximum altitudinal potential was tested using the discrete trait Markov model ARD (all rates different) in the *phytools* R-package[24] using the UCEs plus other molecular markers phylogenetic trees. In total, five altitudinal ranges were considered and species were assigned according to the maximum altitude in which they are found: below 1,000 m; 1,001 to 2,000 m; 2,001 to 3,000 m; 3,001 to 4,000 m; and > 4,001 m. These classifications are inherently subjective to some degree. We focused particularly on the

New Guinea landmass, which possess the highest elevations, the most species diversity at high elevations and the most extensive montane habitat in the region. The rationale for the altitudinal categorization was as follows: below 1,000 m, stagnant water habitat suitable for these beetles are confined to lowland swamps, inundation forest, and riverbank puddles; the range from 1,001 to 2,000 m includes higher intramontane depressions or valley complexes featuring lake-associated and riverine wetlands; the 2,001 to 3,000 m range generally offers fewer suitable habitats, some depressions close to 3,000 m feature small peat swamps and inundated forest; the 3,001 to 4,000 m band includes extensive stretches of alpine meadows and peat swamps; finally, habitat above 4,001 m are limited and poorly explored, characterized by rocky or gravelly pools in the alpine zone.

Habitat was also reconstructed considering whether species are found in subterranean, interstitial or epigean habitats. The colonization of groundwater has previously been studied[25,26], leading to a likely independent transition and a very limited intra-calcrete speciation (i.e., potential sympatric speciation, although limited micro-allopatry within the calcrete could still take place due to fluctuations of the water table). Although the calcretes in which species live are isolated, their spatial connectivity may have been different in the past, and thus, our model allows dispersal among calcretes in the same palaeovalleys. This assumption is supported by recent analyses of speciation in subterranean beetles, which identified several related species in adjacent calcretes following colonization by the same stygobiotic (i.e., obligate subterranean aquatic) ancestor[27]. For this analysis, we used a custom evolutionary transition matrix of discrete habitat evolution in the *phytools* R-package as before. In total, our sampling contained groundwater species from six different palaeovalleys: Carey, Carnegie, Moore, Murchinson, Nabberu, and Raeside. Three additional states were included for the epigean and interstitial species, the latter considering the two palaeovalleys in which they are found. The custom matrix considers that transitions from interstitial to subterranean habitats are only possible within the same palaeovalley as previously suggested for the syntopic genus *Paroster*[27].

In both cases, ancestral character mapping was then simulated using the *make.simmap* function in the r-package *phytools*[24], considering 1,000 simulations using the consensus topology. We forced the root state during the simulations to be an epigean species below 2,000 m due to the unavailability of higher elevations in the area during the emergence of *Limbodessus* and the unlikely subterranean origin. The adaptations in *Limbodessus* are highly specialized for subterranean environments, including extreme morphological changes such as loss of pigmentation, reduction or absence of vision[28] and wings[29], and specialized metabolic processes[30,31] (see *Life underground* in the Discussion), making reversal to epigean habitat very unlikely, as these traits are typically irreversible and maladaptive in surface environments.

Evolutionary trajectory of phototransduction genes

Annotated reference sequences from the surface species *Allodessus bistrigatus* for the phototransduction genes arrestins 1 and 2, long-wavelength opsin (*lwop*), UVopsin, ciliary Opsin (*cOpsin*), *ninaC*, transient receptor potential (*Trp*) and transient receptor potential-like (*Trp-like*), were retrieved from Langille *et al*.[10] and Tierney *et al*.[32]. The reference sequences were used to blast[9] the newly sequenced whole-genome sequencing libraries. All identified exons for these genes were extracted and aligned using MAFFT auto algorithm[33], and compared to genomic data from these genes generated by Langille *et al*.[10]. Geneious v10.2.6[34] was used to visualize alignments and translate exons, and compare sequence data from phylogenetic sister species to detect shared deleterious mutations (i.e., frameshift and premature stop codons) affecting their reading frame (i.e., affecting the protein structure; Supplementary Information, Table S3). No shared deleterious mutations were detected for the genes *Trp*, *ninaC*, *UVopsin* and *cOpsin*, though limited data for comparison were obtained from the latter 3 genes.

Ecological niche modelling

Maxent v3.4.1[35] was used to develop ecological niche modelling using the *bioclim* dataset[36] together with presence-only data collected by the authors. As we intended to highlight the present-day potential distribution of some *Limbodessus* species, we used default parameters.

Supplementary Results and Discussion

Preliminary phylogenetic analyses

The UCEs datasets, employing alternative completeness thresholds for *Limbodessus*, consistently supported nearly identical topologies. These datasets ranged from 2,350 UCEs (alignment of 1,280,432 bp) to 491 UCEs (alignment of 293,631 bp) at completeness threshold of 50-70% respectively. Therefore, a 50% completeness threshold was considered from further analyses (Supplementary Information, Fig. S2 for the 50% completeness topology).

Analyzes selection

For both the Dytiscidae and *Limbodessus* datasets, analyzes comparison and selection relied on Path sampling / Stepping-stone sampling scores. In the Dytiscidae dataset, regardless of the calibration prior, the uncorrelated relaxed lognormal prior and the best evolutionary models was preferred over the simpler HKY+G+I model (Supplementary App Information endix, Table S6). The most suitable fossil calibration prior relied on the exponential prior.

Bayesian analyzes for *Limbodessus* supported again the uncorrelated relaxed lognormal clock under the best evolutionary models (Supplementary Information, Table S7). The selected configuration was the one for which all loci were analyzed together in PartitionFinder2.

Phylogenetic consideration of *Limbodessus*

Our phylogenetic analyses for *Limbodessus* and related genera provide a robust analysis, identifying *Limbodessus* as monophyletic with high support, with *Allodessus* as its sister genus. This result contrasts with previous studies by Cooper *et al*.[28] and Leijs *et al*.[26], which grouped *Allodessus* with *Limbodessus*, but agrees with Balke and Ribera[37]. Our broad sampling, including species of *Limbodessus* across its entire distributional range, a more complete selection of outgroups, and the use of comprehensive nuclear data, provided insights into the relationships of genera within Bidessini.

The common ancestor for *Limbodessus* and *Allodessus* began diverging in the early Miocene. Subsequently, the *Limbodessus* lineage experienced an initial divergence, dividing species into two clades: one primarily comprising Australian species, and the other consisting mainly of New Guinean and Pacific species. The former includes all the groundwater species, while the latter includes all the alpine species.

Biogeographic evolution

Our comparisons of hypotheses revealed an increased likelihood when using dispersal matrixes, with the most likely scenario being a New Guinea origin without relaxation (i.e., hypothesis M1, with the proto-Papuan archipelago originated c. 15 Ma, Figure 2, Supplementary Information, Figure S3). Across all hypotheses, the DEC and DEC + *J* models were the most suitable for our dataset (Supplementary Information, Table S8), while the remaining models provided marginal AIC weight values (Supplementary Information, Table S8). The main differences between DEC and DEC + *J* models lies in the reconstruction of basal nodes, reconstructed to be widespread ancestors inhabiting most of the geographical area except East Asia (DEC) or New Zealand (DEC + *J*) (Figure 2, Supplementary Information, Figures S3-S5). In any case, BioGeoBEARS estimated the ancestral areas with high confidence (Supplementary Information, Figures S3, S5), and the ancestor of *Limbodessus* was undoubtedly linked to the Sahul continent and the Pacific archipelagos, despite the area being partially submerged at that time[22].

Ancestral altitude potential and habitat reconstruction

The custom evolutionary transition matrix using the ARD model reflects the predicted transition from epigean to subterranean habitats, with interstitial habitats representing a transition state in-between (Supplementary Information, Figure S6). Generally, transitions to subterranean habitats occurred at very low rates, with accelerated transitions once the interstitial habitats were reached.

Additionally, the constrained root for the reconstruction of ancestral altitude potential reflects the transition from lowland to high altitudes (Supplementary Information, Figure S7). Transition rates showed an accelerated shift to high altitudes, although the highest elevation category exhibited a high probability of reversal. This may be explained by the harsh environmental conditions at extreme elevations[38].

Evolutionary trajectory of phototransduction genes

Our analyzes highlighted that at least 18 speciation events may have occurred within the same palaeovalleys (Fig. 1). In addition, we detected 10 speciation events that likely occurred within the same calcrete aquifers. Interestingly, shared deleterious mutations in phototransduction genes were observed for five pairs of sister species inhabiting the same aquifers and one pair of sister species inhabiting adjacent calcretes of the same palaeovalley. Despite substantial missing data due to the different data sources used (i.e., whole-genome sequencing and transcriptomes), we obtained a moderate amount of exonic sequences for the arrestin 1 and 2 genes, *lwop* and *Trp-like*. These were compared with sequence data from a previous study of these phototransduction genes in subterranean and surface *Limbodessus*[10]. We detected several common mutations in these vision-related genes in sister species pairs that affected greatly protein structures through modification of the reading frame or by premature stop codons (Supplementary Information, Table S3). Moreover, we also identified shared deleterious mutations of the arrestin genes in sister species inhabiting adjacent calcretes (i.e., *Limbodessus leysi* and *Limbodessus windarraensis*). These shared mutations affecting vision-related genes in sister species suggested their origin predates speciation, indicating their common ancestor was already adapted to subterranean life (i.e., it was a stygobiont). This further supports the hypothesis that for 10 pairs of species, speciation likely occurred in sympatry within calcretes in the presence of gene flow among diverging lineages. Our results do not support directional selection in phototransduction genes. Nonetheless, a complete overview of the contributions of neutral mutation, natural selection, phenotypic plasticity or epigenetics is necessary before ruling out any option[39].

Fig. S1. Beast maximum clade credibility tree for Dytiscidae family. Posterior probabilities > 0.95 are shown as circles in nodes. Red circles represent calibration points.

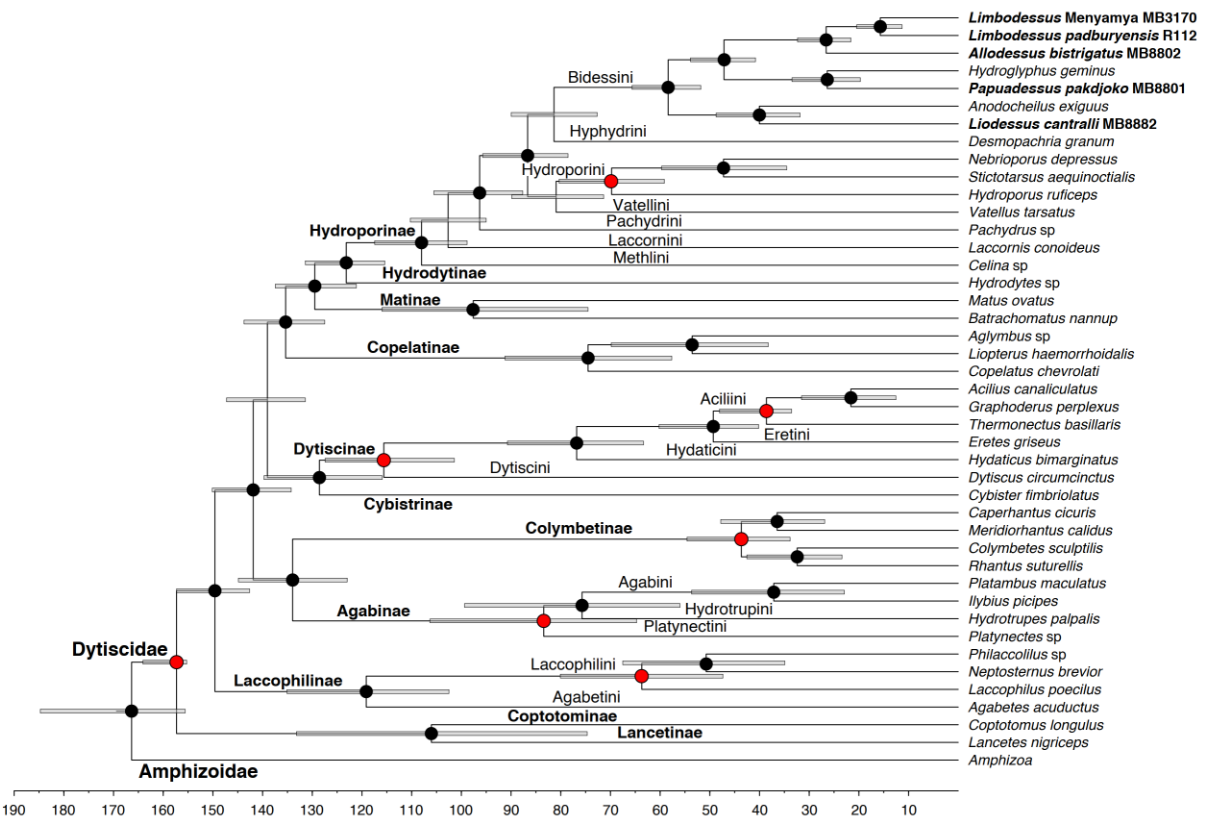

Fig. S2. Beast maximum clade credibility tree for *Limbodessus* including all outgroups. Posterior probabilities > 0.95 are shown as circles in nodes. Red circles represent calibration points.

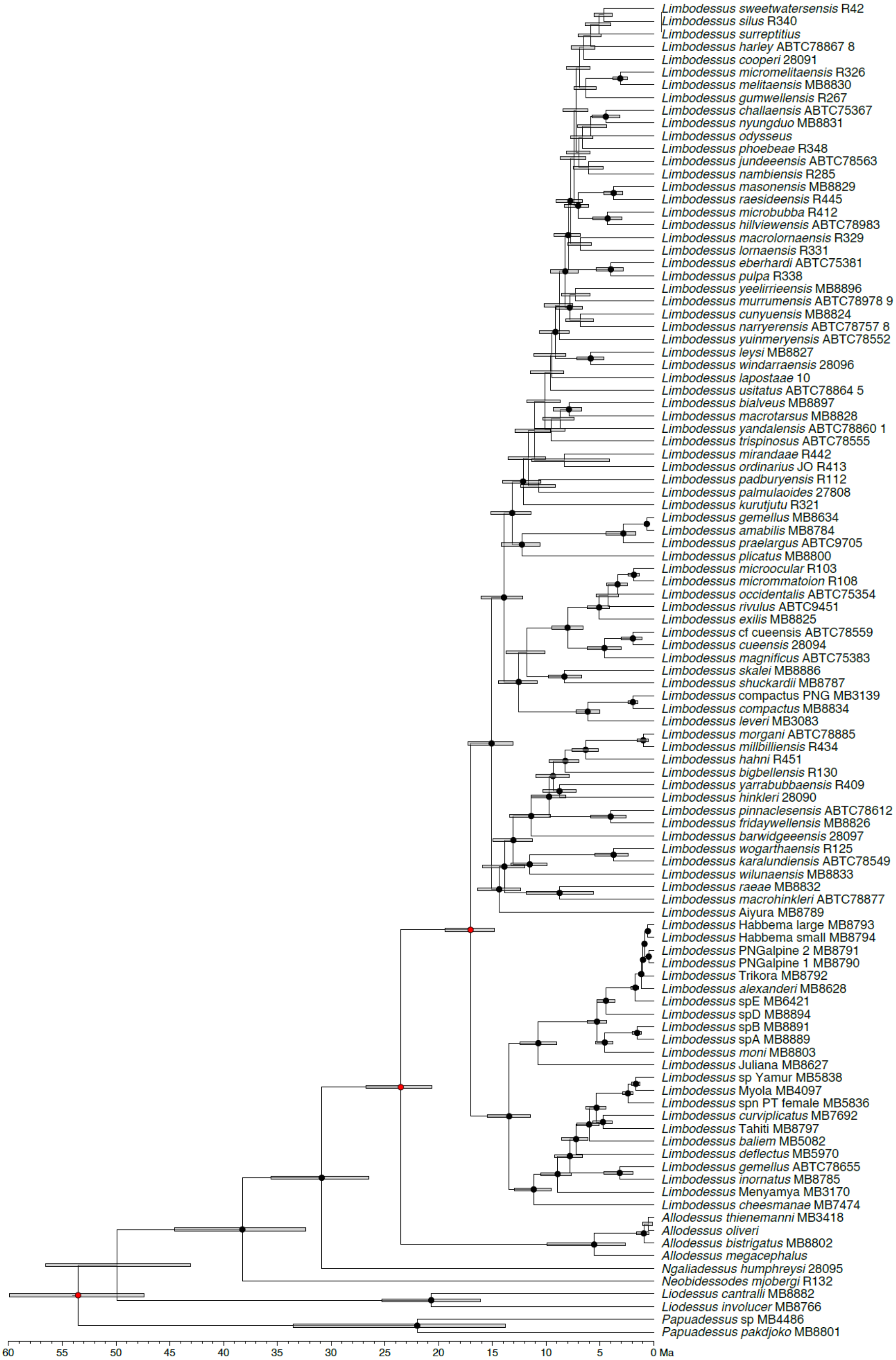

Fig. S3. Biogeographical reconstruction on *Limbodessus* for the hypotheses M1 and DEC + *J* model. Nodes represent the estimated probability for each of the geographical areas: East Asia (A), Australia (B), New Guinea (C), Pacific archipelagos (D) and New Zealand (E). Node colors represent areas as defined in the terminal nodes.

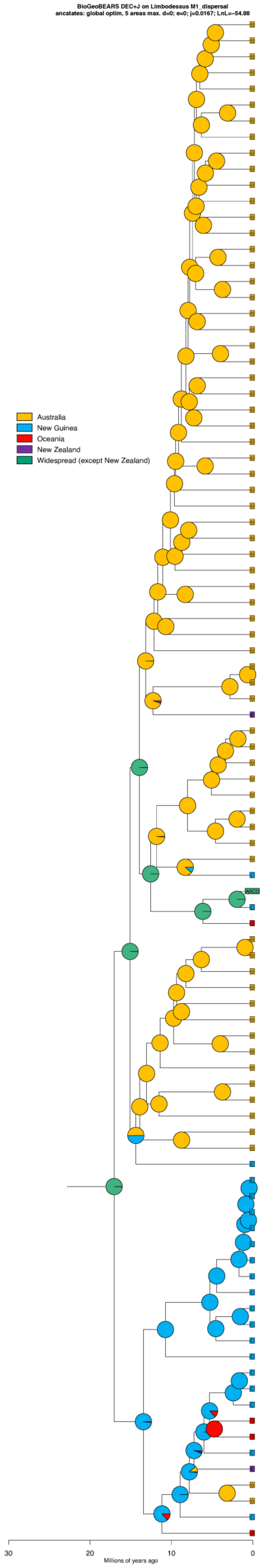

Fig. S4. Biogeographical reconstruction on *Limbodessus* for the hypotheses M1 and DEC model. Nodes represent the estimated geographical areas: East Asia (A), Australia (B), New Guinea (C), Pacific archipelagos (D) and New Zealand (E).

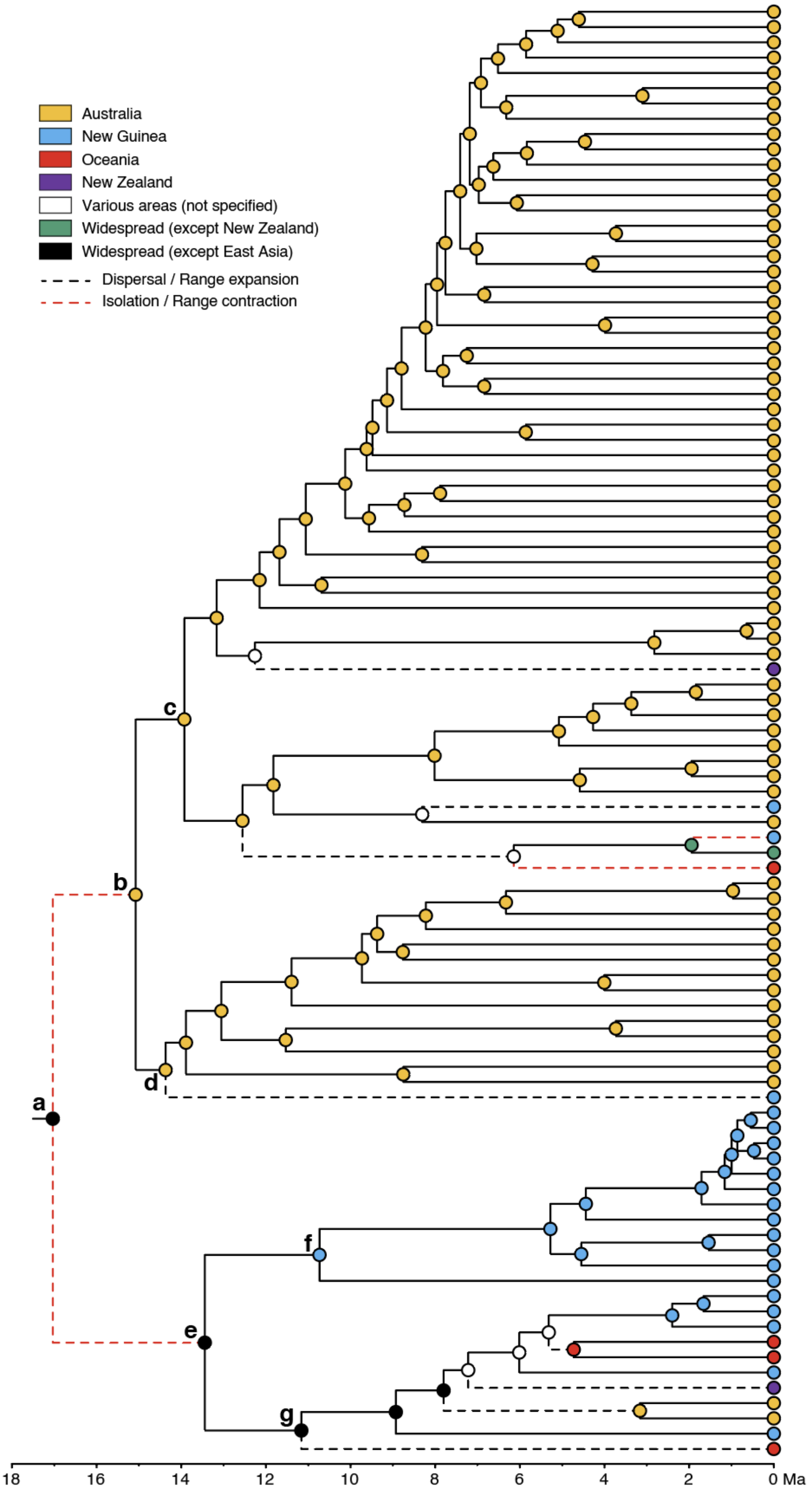

Fig. S5. Biogeographical reconstruction on *Limbodessus* for the hypotheses M1 and DEC model. Nodes represent the estimated probability for each of the geographical areas: East Asia (A), Australia (B), New Guinea (C), Pacific archipelagos (D) and New Zealand (E). Node colors represent areas as defined in the terminal nodes.

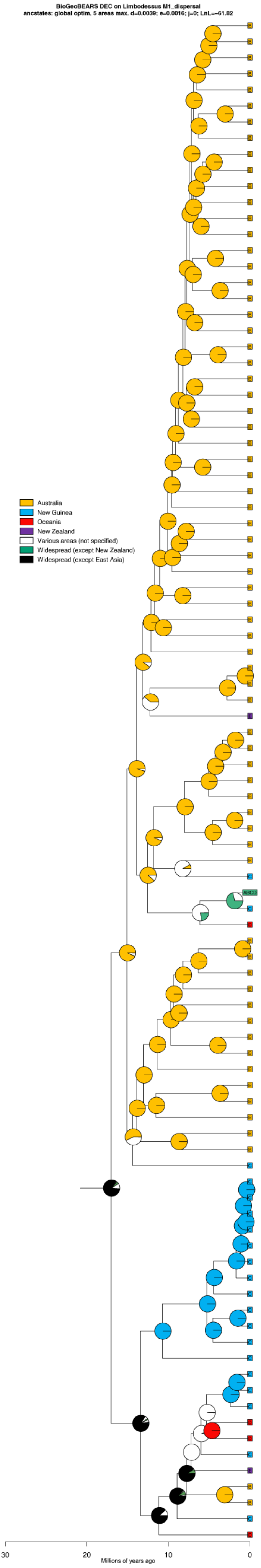

Fig. S6. Fitted All Rates Different (ARD) model for the ancestral habitat reconstruction.

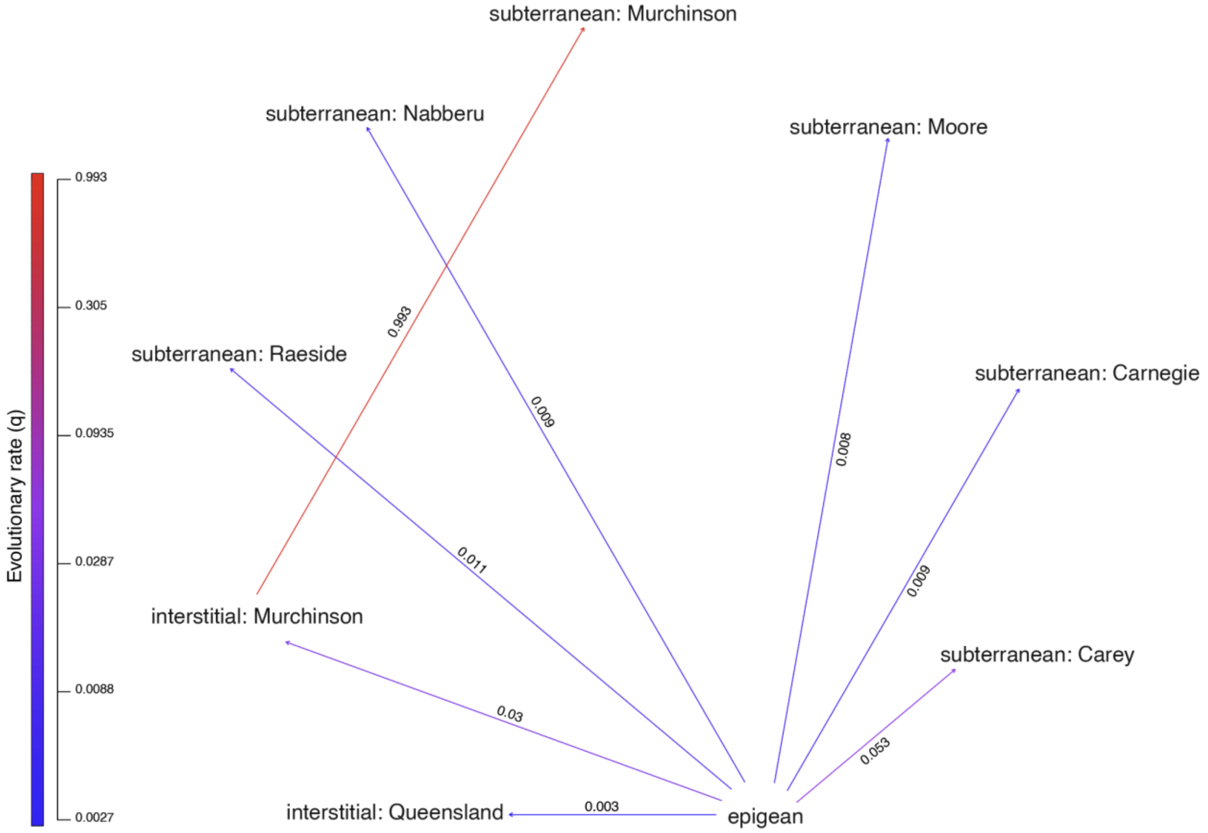

Fig. S7. Fitted All Rates Different (ARD) model for the ancestral altitude potential reconstruction.

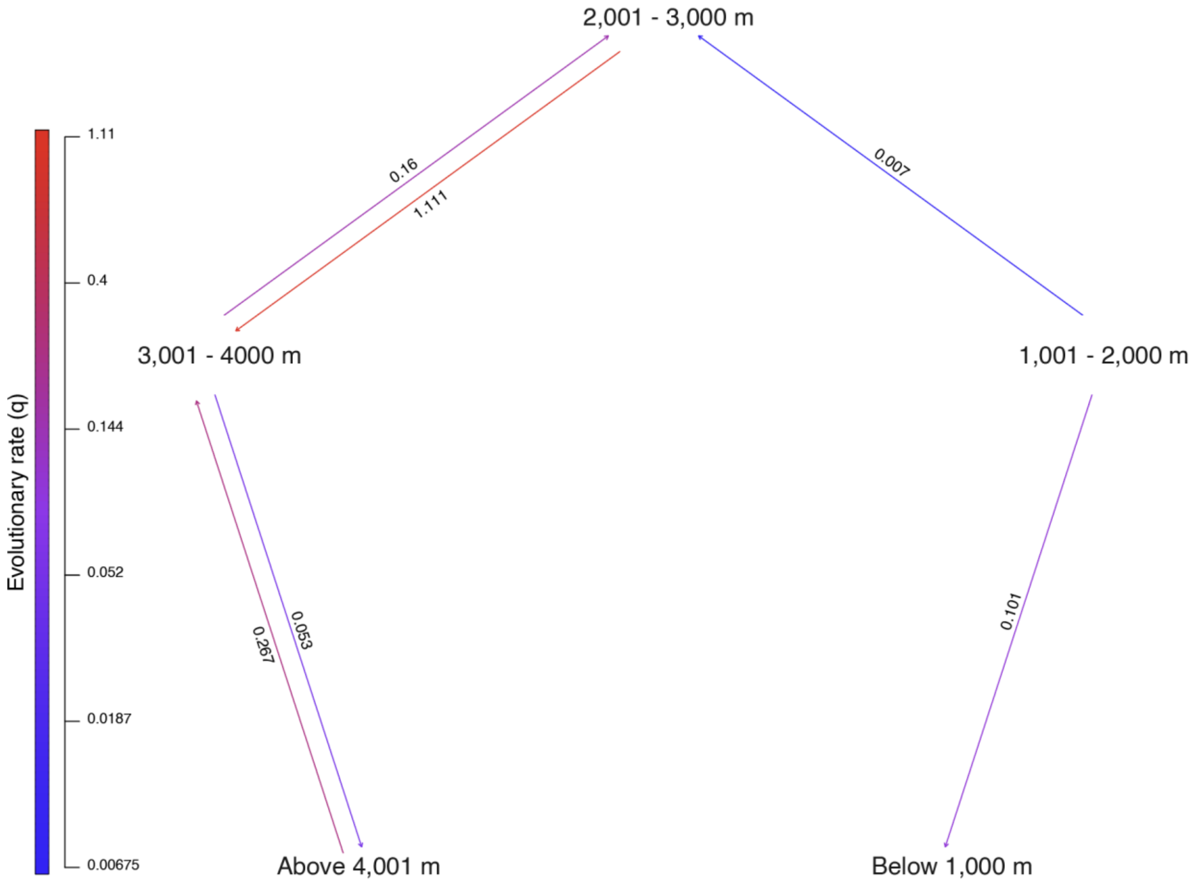

Table S1. List of material used in the dataset for the Dytiscidae family, including taxonomic information, voucher information if available and accession number. In bold, newly sequenced data.

Table S1. List of material used in the dataset for the Dytiscidae family, including taxonomic information, voucher information if available and SRA accession data. In bold, newly sequenced data.

Family	Subfamily	Tribe	Genera	Species	Voucher	Accession number
Dytiscidae	Hydroporinae	Bidessini	*Limbodessus*	sp	MB3170	**SAMN43298430**
Dytiscidae	Hydroporinae	Bidessini	*Limbodessus*	*padburyensis*	R112	**SAMN43298434**
Dytiscidae	Hydroporinae	Bidessini	*Allodessus*	*bistrigatus*	MB8802	**SAMN43298473**
Dytiscidae	Hydroporinae	Bidessini	*Hydroglyphus*	*geminus*	-	SAMN15489339
Dytiscidae	Hydroporinae	Bidessini	*Papuadessus*	*pakdjoko*	MB8801	**SAMN43298474**
Dytiscidae	Hydroporinae	Bidessini	*Anodocheilus*	*exiguus*	SLE1691	SAMN13040641
Dytiscidae	Hydroporinae	Bidessini	*Liodessus*	*cantralli*	MB8882	**SAMN43298471**
Dytiscidae	Hydroporinae	Hyphydrini	*Desmopachris*	*granum*	SLE1690	SAMN13040640
Dytiscidae	Hydroporinae	Hydroporini	*Nebrioporus*	*depressus*	AAU-123	SAMN14403766
Dytiscidae	Hydroporinae	Hydroporini	*Stictotarsus*	*aequinoctialis*	-	SAMN02313993
Dytiscidae	Hydroporinae	Hydroporini	*Hydroporus*	*ruficeps*	SLE1689	SAMN13040639
Dytiscidae	Hydroporinae	Vatellini	*Vatellus*	*tarsatus*	SLE1771	SAMN13040656
Dytiscidae	Hydroporinae	Pachydrini	*Pachydrus*	sp	SLE1763	SAMN13040653
Dytiscidae	Hydroporinae	Laccornini	*Laccornis*	*conoideus*	KBM79	SAMN13040610
Dytiscidae	Hydroporinae	Methlini	*Celina*	sp	SLE1770	SAMN13040655
Dytiscidae	Hydrodytinae	-	*Hydrodytes*	sp	SLE1723	SAMN13040649
Dytiscidae	Matinae	Matini	*Matus*	*ovatus*	SLE1687	SAMN13040637
Dytiscidae	Matinae	Matini	*Batrachomatus*	*nannup*	-	SAMN07280954
Dytiscidae	Copelatinae	Copelatini	*Aglymbus*	sp	SLE935	SAMN13040613
Dytiscidae	Copelatinae	Copelatini	*Liopterus*	*haemorrhoidalis*	-	SAMN07280875
Dytiscidae	Copelatinae	Copelatini	*Copelatus*	*chevrolati*	SLE1688	SAMN13040638
Dytiscidae	Dytiscinae	Aciliini	*Acilius*	*canaliculatus*	-	SAMN15489351
Dytiscidae	Dytiscinae	Aciliini	*Graphoderus*	*perplexus*	SLE1682	SAMN13040632
Dytiscidae	Dytiscinae	Aciliini	*Thermonectus*	*basillaris*	-	SAMN15489320
Dytiscidae	Dytiscinae	Eretini	*Eretes*	*griseus*	-	SAMN15489383
Dytiscidae	Dytiscinae	Hydaticini	*Hydaticus*	*bimargiantus*	SLE1685	SAMN13040635
Dytiscidae	Dytiscinae	Dytiscini	*Dytiscis*	*circumcinctus*	SLE1684	SAMN13040634
Dytiscidae	Cybistrinae	Cybistrini	*Cybister*	*fimbriolatus*	SLE1698	SAMN13040643
Dytiscidae	Colymbetinae	Colymbetini	*Caperhantus*	*cicuris*	-	SAMN15489311
Dytiscidae	Colymbetinae	Colymbetini	*Meridiorhantus*	*calidus*	-	SAMN15489394
Dytiscidae	Colymbetinae	Colymbetini	*Colymbetes*	*sculptilis*	SLE1680	SAMN13040630
Dytiscidae	Colymbetinae	Colymbetini	*Rhantus*	*surreptillis*	SLE1678	SAMN13040628
Dytiscidae	Agabinae	Agabini	*Platambus*	*maculatus*	-	SAMN15489367
Dytiscidae	Agabinae	Agabini	*Illybius*	*picipes*	SLE1683	SAMN13040633
Dytiscidae	Agabinae	Hydrotrupini	*Hydrotrupes*	*palpalis*	-	SAMN15489312
Dytiscidae	Agabinae	Platynectini	*Platynectes*	sp	SLE956	SAMN13040614
Dytiscidae	Laccophilinae	Laccophilini	*Philaccolilus*	sp	-	SAMN15489381
Dytiscidae	Laccophilinae	Laccophilini	*Neptosternus*	*brevior*	-	SAMN15489307
Dytiscidae	Laccophilinae	Laccophilini	*Laccophilus*	*poecilus*	-	SAMN15489358
Dytiscidae	Laccophilinae	Agabetini	*Agabetes*	*acuductus*	KBM80	SAMN13040611
Dytiscidae	Coptotominae	Coptotomini	*Coptotomus*	*longulus*	SLE1686	SAMN13040636
Dytiscidae	Lancetinae	Lancetini	*Lancetes*	*nigriceps*	KBM52	SAMN13040609
Amphizoidae	-	-	*Amphizoa*	sp	-	SAMN10834266

Table S2. List of material used in the *Limbodessus* dataset, including taxonomic information, voucher information, locality data and accession numbers for various molecular markers.

Genera	Species	Voucher	Country	Locality	Year	SRA	cox1	rrnL	mtgenomes	5.8S	18S	28S	H1	H2A	H2B	H3	H4	arginine kinase	topoisomerase	wingless
Allodessus	*bistrigatus*	MB8802	Australia	Canberra	2015	**SAMN43298473**			OQ526059	**PQ129635**	**PQ129560**	**PQ129712**		**PQ123671**	**PQ123599**	**PQ123525**	**PQ123450**	**PQ123227**	**PQ123307**	**PQ123381**
Allodessus	*megacephalus*						AY368227	AY368223												
Allodessus	*oliveri*						AY368228	AY368224												
Allodessus	*thienemanni*	MB3418					FN391941													
Neobidessodes	*mjobergi*	R132	Australia						**PQ197328**											
Ngaliadessus	*humphreysi*	BES18753.1	Australia						OQ526061	**PQ129710**	OQ512863	**PQ129787**	**PQ124962**	**PQ123742**	**PQ123667**	OQ476228	**PQ123516**			
Liodessus	*cantralli*	MB8882	Canada		1973	**SAMN43298471**			**PQ197326**	**PQ129708**	**PQ129632**	**PQ129785**	**PQ124960**	**PQ123740**	**PQ123665**	**PQ123591**	**PQ123515**	**PQ123304**		
Liodessus	*involucer*	MB8766	United Kingdom	Tristan da Cunha		**SAMN43298427**			**PQ197327**	**PQ129709**	**PQ129633**	**PQ129786**	**PQ124961**	**PQ123741**	**PQ123666**	**PQ123592**		**PQ123305**		**PQ123449**
Papuadessus	*pakdjoko*	MB8801	Indonesia	Papua, Sarmi, Waaf, N Foja Mts, riverbank, 120m	2014	**SAMN43298474**			**PQ197329**	**PQ129711**	**PQ129634**	**PQ129788**	**PQ124963**	**PQ123743**	**PQ123668**	**PQ123523**	**PQ123517**	**PQ123306**		
Papuadessus	sp	MB4486					HG327112	HG327113												
Limbodessus	*alexanderi*	MB8628	Indonesia						**PQ197257**	**PQ129636**	**PQ129561**	**PQ129713**	**PQ124899**	**PQ123672**	**PQ123600**	**PQ123526**	**PQ123452**			
Limbodessus	*amabilis*	MB8784	Australia	N TAS, Cradle Valley. Ronny Creek, Boardwalk, 871 m. peat boogs	2006	**SAMN43298450**			**PQ197258**	**PQ129637**	**PQ129562**	**PQ129714**		**PQ123673**	**PQ123601**	**PQ123527**	**PQ123452**	**PQ123228**	**PQ123308**	**PQ123382**
Limbodessus	*baliem*	MB5082	Indonesia	Wamena, 20 mins towd Jiwika, limestone creek	2011	**SAMN43298448**			**PQ197259**	**PQ129638**	**PQ129563**	**PQ129715**	**PQ124900**	**PQ123674**	**PQ123602**	**PQ123528**		**PQ123229**	**PQ123343**	**PQ123383**
Limbodessus	*barwidgeeensis*	28097	Australia						**PQ197260**	**PQ129639**	**PQ129564**	**PQ129716**		**PQ123675**	**PQ123603**	**PQ123529**	**PQ123453**	**PQ123230**	**PQ123309**	
Limbodessus	*bialveus*	MB8897	Australia	Cunyu SBF	2018	**SAMN43298467**			**PQ197261**	**PQ129640**	**PQ129565**	**PQ129717**	**PQ124901**	**PQ123676**	**PQ123604**	**PQ123530**	**PQ123454**	**PQ123231**	**PQ123344**	**PQ123384**
Limbodessus	*bigbellensis*	R130	Australia	Lake Austin	2002	**SAMN43298452**			**PQ197262**	**PQ129641**	**PQ129566**	**PQ129718**	**PQ124902**	**PQ123677**	**PQ123605**	**PQ123531**	**PQ123455**	**PQ123232**	**PQ123345**	**PQ123385**
Limbodessus	*challaensis*	ABTC75367	Australia				AF484142	AF485947										**PQ123233**		
Limbodessus	*cheesmanae*	MB7474	New Caledonia		2016	**SAMN43298447**			**PQ197263**	**PQ129642**	**PQ129567**	**PQ129719**	**PQ124903**	**PQ123678**	**PQ123606**	**PQ123532**	**PQ123456**	**PQ123234**	**PQ123346**	**PQ123386**
Limbodessus	*compactus*	MB8834	Australia	Garradunga		**SAMN43298472**			OP168264	**PQ129643**	**PQ129568**	**PQ129720**		**PQ123679**	**PQ123593**	**PQ123533**	**PQ123457**	**PQ123235**	**PQ123310**	**PQ123387**
Limbodessus	*cooperi*	28091	Australia						**PQ197264**	**PQ129644**	**PQ129569**	**PQ129721**	**PQ124904**	**PQ123680**	**PQ123594**	**PQ123534**	**PQ123458**	**PQ123236**	**PQ123311**	**PQ123388**
Limbodessus	*cueensis*	28094	Australia						**PQ197265**	**PQ129645**	**PQ129570**	**PQ129722**	**PQ124905**	**PQ123681**	**PQ123607**	**PQ123535**	**PQ123459**	**PQ123237**	**PQ123312**	**PQ123389**
Limbodessus	cf. *cueensis*	ABTC78559	Australia				AY350888	AY353835												
Limbodessus	*cunyuensis*	MB8824	Australia	Sweetwaters Well	2007	**SAMN43298445**			**PQ197266**	**PQ129646**	**PQ129571**	**PQ129723**	**PQ124906**	**PQ123682**	**PQ123608**	**PQ123536**	**PQ123460**	**PQ123238**	**PQ123347**	**PQ123390**
Limbodessus	*curviplicatus*	MB7692	Samoa	NW Pua'pu'a, puddle on gravel w grass mats		**SAMN43298424**			**PQ197267**	**PQ129647**	**PQ129572**	**PQ129724**	**PQ124907**	**PQ123683**	**PQ123609**	**PQ123537**	**PQ123461**			
Limbodessus	*deflectus*	MB5970	New Zealand	Te Urewera NP, Lake Waikaremoana, Waipai swam, 1.6 km NWW Aniwaniwa	2012	**SAMN43298477**			**PQ197268**	**PQ129648**	**PQ129573**	**PQ129725**	**PQ124908**	**PQ123684**	**PQ123595**	**PQ123538**	**PQ123462**	**PQ123239**	**PQ123348**	
Limbodessus	*eberhardi*	ABTC75381	Australia				AF484152	AF485957		**PQ129649**	**PQ129574**	**PQ129726**	**PQ124909**		**PQ123596**	**PQ123518**		**PQ123240**	**PQ123313**	**PQ123391**
Limbodessus	*exilis*	MB8825	Australia	Maranalgo	2019	**SAMN43298444**			**PQ197269**	**PQ129650**	**PQ129575**	**PQ129727**	**PQ124910**	**PQ123685**	**PQ123610**	**PQ123539**	**PQ123463**	**PQ123241**	**PQ123349**	**PQ123392**
Limbodessus	*fridaywellensis*	MB8826	Australia	Depot Springs	2018	**SAMN43298453**			**PQ197270**	**PQ129651**	**PQ129576**	**PQ129728**	**PQ124911**	**PQ123686**	**PQ123611**	**PQ123540**	**PQ123464**	**PQ123242**	**PQ123350**	**PQ123393**
Limbodessus	*gemellus*	ABTC78655	Australia				AY350901	AY353848												
Limbodessus	*gemellus*	MB8634	Australia	S NSW.Eden, Towamba Road 2km N Nullica	2006	**SAMN43298425**			**PQ197271**	**PQ129652**	**PQ129577**	**PQ129729**		**PQ123687**	**PQ123612**	**PQ123541**		**PQ123243**	**PQ123314**	**PQ123394**
Limbodessus	*gumwellensis*	R267	Australia	Gum Well: Perrinvale	2004	**SAMN43298465**			**PQ197272**	**PQ129653**	**PQ129578**	**PQ129730**	**PQ124912**	**PQ123688**	**PQ123613**	**PQ123542**	**PQ123465**	**PQ123244**	**PQ123351**	**PQ123395**
Limbodessus	*hahni*	R451	Australia	Magellan mine: Uramurdah		**SAMN43298462**			**PQ197273**	**PQ129654**	**PQ129579**	**PQ129731**		**PQ123689**	**PQ123597**	**PQ123543**		**PQ123245**	**PQ123352**	**PQ123396**
Limbodessus	*harleyi*	ABTC78867/78868	Australia				JQ745773	JQ745740										**PQ123246**	**PQ123315**	**PQ123397**
Limbodessus	*hillviewensis*	ABTC78983	Australia				JQ745758	JQ745726										**PQ123247**	**PQ123316**	**PQ123398**
Limbodessus	*hinkleri*	28090	Australia						**PQ197274**	**PQ129655**	**PQ129580**	**PQ129732**	**PQ124913**	**PQ123690**	**PQ123614**	**PQ123544**		**PQ123248**	**PQ123317**	**PQ123399**
Limbodessus	*inornatus*	MB8785	Australia	WA, Bridgetown. Pools near Blackwood River	2015	**SAMN43298475**			**PQ197275**	**PQ129656**	**PQ129581**	**PQ129733**	**PQ124914**	**PQ123691**	**PQ123615**	**PQ123545**		**PQ123249**		**PQ123400**
Limbodessus	*jundeeensis*	ABTC78563	Australia				AY350887	AY353834										**PQ123250**		**PQ123401**
Limbodessus	*karalundiensis*	ABTC78549	Australia				AY350891	AY353838										**PQ123251**		**PQ123402**
Limbodessus	*kurutjutu*	R321	Australia				**PQ216011**											**PQ123252**	**PQ123318**	**PQ123403**
Limbodessus	*lapostaae*	10	Australia						**PQ197276**	**PQ129657**	**PQ129582**	**PQ129734**	**PQ124915**	**PQ123692**	**PQ123616**	**PQ123546**	**PQ123466**	**PQ123253**	**PQ123319**	**PQ123404**
Limbodessus	*leveri*	MB3083	Fiji	Vanua Levu: 5km N Savusavu	2003	**SAMN43298449**			**PQ197277**	**PQ129658**	**PQ129583**	**PQ129735**		**PQ123693**	**PQ123617**	**PQ123547**	**PQ123467**	**PQ123254**	**PQ123353**	**PQ123405**
Limbodessus	*leysi*	MB8827	Australia	Mt Morgans Borefield	2018	**SAMN43298454**			**PQ197278**	**PQ129659**	**PQ129584**	**PQ129736**	**PQ124916**	**PQ123694**	**PQ123618**	**PQ123548**	**PQ123468**	**PQ123255**	**PQ123320**	**PQ123406**
Limbodessus	*lornaensis*	R331	Australia	Lorna Glen Station	2005	**SAMN43298483**			**PQ197279**	**PQ129660**	**PQ129585**	**PQ129737**	**PQ124917**	**PQ123695**	**PQ123619**	**PQ123549**	**PQ123469**	**PQ123256**	**PQ123321**	**PQ123407**
Limbodessus	*microhinkleri*	ABTC78877	Australia				JQ745765	JQ745733												
Limbodessus	*macrolornaensis*	R329	Australia	Lorna Glen Station	2005	**SAMN43298464**			**PQ197280**	**PQ129661**	**PQ129586**	**PQ129738**	**PQ124918**	**PQ123696**	**PQ123620**	**PQ123550**	**PQ123470**	**PQ123257**	**PQ123322**	**PQ123408**
Limbodessus	*macrotarsus*	MB8828	Australia	Cunyu SBF	2018	**SAMN43298455**			**PQ197281**	**PQ129662**	**PQ129587**	**PQ129739**		**PQ123669**	**PQ123621**	**PQ123519**	**PQ123471**	**PQ123258**	**PQ123354**	**PQ123409**
Limbodessus	*magnificus*	ABTC75383	Australia				AF484149	AF485954		**PQ129663**		**PQ129740**						**PQ123259**	**PQ123323**	**PQ123410**
Limbodessus	*masonensis*	MB8829	Australia	Lake Mason	2011	**SAMN43298456**			**PQ197282**	**PQ129664**	**PQ129588**	**PQ129741**	**PQ124919**	**PQ123697**	**PQ123622**	**PQ123520**	**PQ123472**	**PQ123260**	**PQ123355**	**PQ123411**
Limbodessus	*melitaensis*	MB8830	Australia	Melita	2018	**SAMN43298429**			**PQ197283**	**PQ129665**	**PQ129589**	**PQ129742**	**PQ124920**	**PQ123698**	**PQ123623**	**PQ123551**	**PQ123473**	**PQ123261**	**PQ123356**	**PQ123412**
Limbodessus	*microbubba*	R412	Australia	Yarrabubba Station: North	2005	**SAMN43298481**			**PQ197284**	**PQ129666**	**PQ129590**	**PQ129743**	**PQ124921**	**PQ123699**	**PQ123624**	**PQ123552**	**PQ123474**	**PQ123262**	**PQ123357**	**PQ123413**
Limbodessus	*micromelitaensis*	R326	Australia	Melita Station	2005	**SAMN43298461**			**PQ197285**	**PQ129667**	**PQ129591**	**PQ129744**	**PQ124922**	**PQ123700**	**PQ123625**	**PQ123553**	**PQ123475**	**PQ123263**	**PQ123358**	**PQ123414**
Limbodessus	*micrommatoion*	R108	Australia						**PQ197286**	**PQ129668**	**PQ129592**	**PQ129745**	**PQ124923**	**PQ123701**	**PQ123626**	**PQ123554**	**PQ123476**	**PQ123264**	**PQ123324**	**PQ123415**
Limbodessus	*microocula*	R103	Australia						**PQ197287**	**PQ129669**	**PQ129593**	**PQ129746**	**PQ124924**	**PQ123702**	**PQ123627**	**PQ123555**	**PQ123477**	**PQ123265**	**PQ123325**	**PQ123416**
Limbodessus	*millbilliensis*	R434	Australia	Bubble Well: Bubble	2006	**SAMN43298480**			**PQ197288**	**PQ129670**	**PQ129594**	**PQ129747**	**PQ124925**	**PQ123703**	**PQ123628**	**PQ123556**	**PQ123478**	**PQ123266**	**PQ123326**	**PQ123417**

Limbodessus	*mirandaae*	R442	Australia	Lake Miranda West	2006	**SAMN43298431**			**PQ197289**	**PQ129671**	**PQ129595**	**PQ129748**	**PQ124926**	**PQ123704**	**PQ123629**	**PQ123557**	**PQ123479**	**PQ123267**	**PQ123359**	**PQ123418**
Limbodessus	*moni*	MB8803	Indonesia			**SAMN43298436**			**PQ197290**	**PQ129672**	**PQ129596**	**PQ129749**	**PQ124927**	**PQ123705**	**PQ123630**	**PQ123558**	**PQ123480**	**PQ123268**	**PQ123360**	
Limbodessus	*morgani*	ABTC78885	Australia				JQ745786	JQ745752										**PQ123269**	**PQ123327**	**PQ123419**
Limbodessus	*murrumensis*	ABTC78978/78979	Australia				JQ745783	JQ745749										**PQ123270**	**PQ123328**	**PQ123420**
Limbodessus	*nambiensis*	R285	Australia	Nambi	2004	**SAMN43298432**			**PQ197291**	**PQ129673**	**PQ129597**	**PQ129750**	**PQ124928**	**PQ123706**	**PQ123631**	**PQ123559**	**PQ123481**	**PQ123271**	**PQ123361**	**PQ123421**
Limbodessus	*narryerensis*	ABTC78757/78758	Australia				JQ745761	JQ745729										**PQ123272**	**PQ123329**	**PQ123422**
Limbodessus	*nyungduo*	MB8831	Australia	Challa North	2019	**SAMN43298433**			**PQ197292**	**PQ129674**	**PQ129598**	**PQ129751**	**PQ124929**	**PQ123707**	**PQ123632**	**PQ123560**	**PQ123482**	**PQ123273**	**PQ123362**	**PQ123423**
Limbodessus	*occidentalis*	ABTC75354	Australia				AF484150	AF485955										**PQ123274**	**PQ123330**	**PQ123424**
Limbodessus	*odysseus*		Australia				KU549165													
Limbodessus	*ordinarius*	R413	Australia															**PQ123275**		**PQ123425**
Limbodessus	*padburyensis*	R112	Australia	Mt Padbury	2002	**SAMN43298434**			**PQ197293**	**PQ129675**	**PQ129599**	**PQ129752**	**PQ124930**	**PQ123708**	**PQ123633**	**PQ123561**	**PQ123483**	**PQ123276**	**PQ123363**	**PQ123426**
Limbodessus	*palmulaoides*	27808	Australia						**PQ197294**	**PQ129676**	**PQ129600**	**PQ129753**	**PQ124931**	**PQ123709**	**PQ123634**	**PQ123562**	**PQ123484**	**PQ123277**	**PQ123331**	**PQ123427**
Limbodessus	*phoebeae*	R348	Australia	Yakabindie Station: Miranda east	2004	**SAMN43298482**			**PQ197295**	**PQ129677**	**PQ129601**	**PQ129754**	**PQ124932**	**PQ123710**	**PQ123635**	**PQ123563**	**PQ123485**	**PQ123278**	**PQ123364**	**PQ123428**
Limbodessus	*pinnaclesensis*	ABTC78612	Australia				AY350899	AY353846										**PQ123279**	**PQ123332**	**PQ123429**
Limbodessus	*plicatus*	MB8800	New Zealand	Mt. Aspiring NP, 11.xii.2012 Pleasan Flat 31 Km SWW of blaast	2012	**SAMN43298440**			**PQ197296**	**PQ129678**	**PQ129602**	**PQ129755**	**PQ124933**	**PQ123670**	**PQ123636**	**PQ123521**	**PQ123486**		**PQ123365**	
Limbodessus	*praelargus*	ABTC9705	Australia				AF484139	AF485944												
Limbodessus	*pulpa*	R338	Australia						**PQ197297**	**PQ129679**	**PQ129603**	**PQ129756**	**PQ124934**	**PQ123711**	**PQ123637**	**PQ123564**	**PQ123487**	**PQ123280**	**PQ123333**	**PQ123430**
Limbodessus	*raeae*	MB8832	Australia	Hinkler Well	2013	**SAMN43298443**			**PQ197298**	**PQ129680**	**PQ129604**	**PQ129757**	**PQ124935**	**PQ123712**	**PQ123638**	**PQ123565**	**PQ123488**	**PQ123281**	**PQ123366**	**PQ123431**
Limbodessus	*raesideensis*	R445	Australia	Salt Well: Lake Mason	2006	**SAMN43298479**			**PQ197299**	**PQ129681**	**PQ129605**	**PQ129758**	**PQ124936**	**PQ123713**	**PQ123639**	**PQ123566**	**PQ123489**	**PQ123282**	**PQ123334**	**PQ123432**
Limbodessus	*rivulus*	ABTC9451	Australia				AF484129	AF485934												**PQ123433**
Limbodessus	*shuckardii*	MB8787	Australia	C QLD, 19 km S Ayr, Bannister Lagoon at Bruce Hwy, swamp. 20m	2006	**SAMN43298428**			**PQ197300**	**PQ129682**	**PQ129606**	**PQ129759**	**PQ124937**	**PQ123714**	**PQ123640**	**PQ123522**	**PQ123490**	**PQ123283**		
Limbodessus	*silus*	R340	Australia	Cunyu: Sweetwaters	2005	**SAMN43298435**			**PQ197301**	**PQ129683**	**PQ129607**	**PQ129760**	**PQ124938**	**PQ123715**	**PQ123641**	**PQ123567**	**PQ123491**	**PQ123284**	**PQ123367**	**PQ123434**
Limbodessus	*skalei*	MB8886	Indonesia						**PQ197302**	**PQ129684**	**PQ129608**	**PQ129761**	**PQ124939**	**PQ123716**	**PQ123642**	**PQ123568**	**PQ123492**			
Limbodessus	sp Aiyura	MB8789	Papua New Guinea	Aiyura	2006	**SAMN43298457**			**PQ197310**	**PQ129685**	**PQ129609**	**PQ129762**	**PQ124940**	**PQ123717**	**PQ123598**	**PQ123569**			**PQ123378**	
Limbodessus	sp compactus PNG	MB3139	Papua New Guinea	Madang town, Cleland Park	2006	**SAMN43298478**			**PQ197303**	**PQ129686**	**PQ129610**	**PQ129763**		**PQ123718**	**PQ123643**	**PQ123570**	**PQ123493**	**PQ123285**	**PQ123379**	**PQ123435**
Limbodessus	sp Habbema large	MB8793	Indonesia	Papua, Lake Habbema, poola, 3200m	2011	**SAMN43298442**			**PQ197314**	**PQ129687**	**PQ129611**	**PQ129764**	**PQ124941**	**PQ123719**	**PQ123644**	**PQ123571**	**PQ123494**	**PQ123286**		
Limbodessus	sp Habbema small	MB8794	Indonesia	Papua, Lake Habbema, poola, 3200m	2011	**SAMN43298438**			**PQ197315**	**PQ129688**	**PQ129612**	**PQ129765**		**PQ123720**	**PQ123645**	**PQ123572**	**PQ123495**	**PQ123287**	**PQ123368**	
Limbodessus	sp Juliana	MB8627	Indonesia	Mt. Juliana area	2006	**SAMN43298484**			**PQ197309**	**PQ129689**	**PQ129613**	**PQ129766**	**PQ124942**	**PQ123721**	**PQ123646**	**PQ123573**	**PQ123496**		**PQ123380**	
Limbodessus	sp Menyamya	MB3170	Papua New Guinea	Menyamya, Mt Inji, deep well	2006	**SAMN43298430**			**PQ197304**	**PQ129690**	**PQ129614**	**PQ129767**	**PQ124943**	**PQ123722**	**PQ123647**	**PQ123574**	**PQ123497**	**PQ123288**	**PQ123369**	**PQ123436**
Limbodessus	sp Myola	MB4097	Papua New Guinea	Myola	2008	**SAMN43298437**			**PQ197305**	**PQ129691**	**PQ129615**	**PQ129768**	**PQ124944**	**PQ123723**	**PQ123648**	**PQ123575**	**PQ123498**		**PQ123370**	**PQ123437**
Limbodessus	sp PNGalpine 1	MB8790	Papua New Guinea	Southern Highlands, Sopulkul, 30-35 km NE Mendi, Ex swamp into stream, 2679 m	2006	**SAMN43298441**			**PQ197311**	**PQ129692**	**PQ129616**	**PQ129769**	**PQ124945**	**PQ123724**	**PQ123649**	**PQ123576**	**PQ123499**		**PQ123371**	
Limbodessus	sp PNGalpine 2	MB8791	Papua New Guinea	Enga, Kumul Lodge @ foot of Mt. Hagen, 2700 m	2006	**SAMN43298446**			**PQ197312**	**PQ129693**	**PQ129617**	**PQ129770**	**PQ124946**	**PQ123725**	**PQ123650**	**PQ123577**	**PQ123500**	**PQ123289**	**PQ123372**	
Limbodessus	sp spA	MB8889	Indonesia	Between Aipomek to Langda	1992	**SAMN43298470**			**PQ197317**	**PQ129694**	**PQ129618**	**PQ129771**	**PQ124947**	**PQ123726**	**PQ123651**	**PQ123578**	**PQ123501**	**PQ123290**		
Limbodessus	sp spB	MB8891	Indonesia	Mt. Elit	1993	**SAMN43298469**			**PQ197318**	**PQ129695**	**PQ129619**	**PQ129772**	**PQ124948**	**PQ123727**	**PQ123652**	**PQ123579**	**PQ123502**	**PQ123291**		
Limbodessus	sp spD	MB8894	Indonesia	Mt. Trikora foot, Baliem River valley (Oue Tal)	1993	**SAMN43298458**			**PQ197319**	**PQ129696**	**PQ129620**	**PQ129773**	**PQ124949**	**PQ123728**	**PQ123653**	**PQ123580**	**PQ123503**			
Limbodessus	sp spE	MB6421	Indonesia	Between Kono, Angguruk	1992	**SAMN43298476**			**PQ197308**	**PQ129697**	**PQ129621**	**PQ129774**	**PQ124950**	**PQ123729**	**PQ123654**	**PQ123581**	**PQ123504**		**PQ123375**	
Limbodessus	sp spn PT female	MB5836	Indonesia	Enarotali, Danau Paniai	1991	**SAMN43298460**			**PQ197306**	**PQ129698**	**PQ129622**	**PQ129775**	**PQ124951**	**PQ123730**	**PQ123655**	**PQ123582**	**PQ123505**		**PQ123373**	
Limbodessus	sp Tahiti	MB8797	French Polynesia			**SAMN43298439**			**PQ197316**	**PQ129699**	**PQ129623**	**PQ129776**	**PQ124952**	**PQ123731**	**PQ123656**	**PQ123583**	**PQ123506**			
Limbodessus	sp Trikora	MB8792	Indonesia	Papua, Lake Habbema, 3400m	2018	**SAMN43298451**			**PQ197313**	**PQ129700**	**PQ129624**	**PQ129777**	**PQ124953**	**PQ123732**	**PQ123657**	**PQ123584**	**PQ123507**			
Limbodessus	sp Yamur	MB5838	Indonesia	Fak Fak, Lake Yamur	1998	**SAMN43298459**			**PQ197307**	**PQ129701**	**PQ129625**	**PQ129778**		**PQ123733**	**PQ123658**	**PQ123585**	**PQ123508**	**PQ123292**	**PQ123374**	
Limbodessus	*surreptitius*		Australia															**PQ123293**	**PQ123335**	**PQ123438**
Limbodessus	*sweetwatersensis*	R42	Australia						**PQ197320**	**PQ129702**	**PQ129626**	**PQ129779**	**PQ124954**	**PQ123734**	**PQ123659**	**PQ123586**	**PQ123509**	**PQ123294**	**PQ123336**	**PQ123439**
Limbodessus	*trispinosus*	ABTC78555	Australia				AY350889	AY353836										**PQ123295**	**PQ123337**	**PQ123440**
Limbodessus	*usitatus*	ABTC78864/78865	Australia				JQ745768	JQ745736										**PQ123296**	**PQ123338**	**PQ123441**
Limbodessus	*wilunaensis*	MB8833	Australia	Lake Uramurdah	2018	**SAMN43298426**			**PQ197321**	**PQ129703**	**PQ129627**	**PQ129780**	**PQ124955**	**PQ123735**	**PQ123660**	**PQ123587**	**PQ123510**	**PQ123297**	**PQ123376**	**PQ123442**
Limbodessus	*windarraensis*	28096	Australia						**PQ197322**	**PQ129704**	**PQ129628**	**PQ129781**	**PQ124956**	**PQ123736**	**PQ123661**	**PQ123588**	**PQ123511**	**PQ123298**	**PQ123339**	**PQ123443**
Limbodessus	*wogarthaensis*	R125	Australia	Wogartha Well: Moorarie	2002	**SAMN43298466**			**PQ197323**	**PQ129705**	**PQ129629**	**PQ129782**	**PQ124957**	**PQ123737**	**PQ123662**	**PQ123524**	**PQ123512**	**PQ123299**	**PQ123377**	**PQ123444**
Limbodessus	*yandalensis*	ABTC78860/78861	Australia				JQ745780	JQ745746										**PQ123300**	**PQ123340**	**PQ123445**
Limbodessus	*yarrabubbaensis*	R409	Australia	Yarrabubba Station: South	2005	**SAMN43298463**			**PQ197324**	**PQ129706**	**PQ129630**	**PQ129783**	**PQ124958**	**PQ123738**	**PQ123663**	**PQ123589**	**PQ123513**	**PQ123301**	**PQ123341**	**PQ123446**
Limbodessus	*yeelirrieensis*	MB8896	Australia	Yeelirrie	2018	**SAMN43298468**			**PQ197325**	**PQ129707**	**PQ129631**	**PQ129784**	**PQ124959**	**PQ123739**	**PQ123664**	**PQ123590**	**PQ123514**	**PQ123302**		**PQ123447**
Limbodessus	*yuinmeryensis*	ABTC78552	Australia				AY350890	AY353837										**PQ123303**	**PQ123342**	**PQ123448**

Table S3. Deleterious mutations in phototransduction genes shared among phylogenetic sister species pairs inhabiting the same isolated aquifers or adjacent calcretes of the same palaeovalley.

Table S3. Deleterious mutations in phototransduction genes shared among phylogenetic sister species pairs inhabiting the same isolated aquifers or adjacent calcretes of the same palaeovalley.

**Sister species**	**Arrestin 1**	**Arrestin 2**	**LWOP**	**Trp-like**
L. melitaensis<br>*L. micromelitaensis*	nf	1 bp deletion in exon 5; frameshift and premature stop codon	305 bp deletion of 5' end of exon 1 and > 2 kb upstream flanking sequence deleted	30 bp deletion in exon 13
L. masonensis<br>*L. raesideensis*	nf	1 bp insertion in exon 1; frameshift and premature stop codon	nf	nf
L. eberhardi<br>*L. pulpa*	nf	nf	Stop codon in exon 3	nf
L. microocular<br>*L. micrommatoion*	nf	nf	17 bp deletion in exon 2; frameshift and premature stop codon	nf
*L. leysi**<br>*L. windarraensis**	4 bp deletion in exon 4, frameshift and premature stop codon	1 bp deletion in exon 1; frameshift and premature stop codon; 24 bp deletion in exon 3	nf	nf

*Phylogenetic sister species living in adjacent calcretes. nf - shared deleterious mutations "not found"; additional sister species revealed in Fig. S2 for which no shared mutations were observed in the data include *L. silus*/*L. sweetwatersensis*, *L. nyungduo*/*L. challaensis*, *L. macrotarsus*/*L. bialveus*, *L. raeae*/*L. macrohinkleri* and *L. cueensis*/*L.magnificus*.

Table S4. List of fossils used for the Dytiscidae analyses.

Table S4. List of fossils used for the Dytiscidae analyses.

**Fossil**	**Geological Age**	**Minimum Age (Ma)**	**Lognormal Prior**	**Exponential Prior**	**Constrained groups**
Hydroporus carstengroehni Balke, Beigel & Hendrich, 2010	Middle Eocene	41.3	x = 20<br>SD = 13.47<br>Offset = 36.3	x = 13.65<br>Offset = 40.95	Crown Hydroporini
Hydrotrupes prometheus Gomez & Daamgard, 2014	Middle Eocene	41.3	x = 20<br>SD = 13.47<br>Offset = 36.3	x = 13.65<br>Offset = 40.95	Crown Agabinae
Copelatus aphroditae Balke, 2003	Middle Eocene	41.3	x = 20<br>SD = 13.47<br>Offset = 36.3	x = 13.65<br>Offset = 40.95	Crown Copelatinae
Colymbetes miocaenicus Ríha, 1974	Middle Eocene	11.6	x = 20<br>SD = 13.47<br>Offset = 6.6	x = 13.65<br>Offset = 11.25	Crown Colymbetinae
Japanolaccophilus baetificus Balke & Hendrich, 2019	Middle Eocene	41.3	x = 20<br>SD = 13.47<br>Offset = 36.3	x = 13.65<br>Offset = 40.95	Crown Laccophilini
Acilius florissantensis Wickham, 1909	Early Oligocene	33.9	x = 20<br>SD = 13.47<br>Offset = 28.9	x = 13.65<br>Offset = 33.55	Crown Acilini
Ambarticus myanmaricus Yang et al., 2019	Upper Cretaceous	98.17	x = 20<br>SD = 13.47<br>Offset = 93.17	x = 13.65<br>Offset = 97.82	Crown Dytiscinae
Paleodytes gutta Ponomarenko, 1987	Upper Jurassic	155.7	x = 20<br>SD = 13.47<br>Offset = 150.7	x = 13.65<br>Offset = 155.28	Crown Dytiscidae

Table S5. Custom manual dispersal multiplier matrices for the standard and relaxed origin of New Guinea.

**Table S5**. Custom manual dispersal multiplier matrices for the standard (Miocene) and relaxed (Oligocene) origin of New Guinea.					
**Standard matrix (Present to 15 Ma)**					
	East Asia	Australia	New Guinea	Pacific	New Zealand
East Asia	1	0.5	1	0.1	0.1
Australia	0.5	1	1	0.5	0.5
New Guinea	1	1	1	1	0.1
Pacific	0.1	0.5	1	1	0.5
New Zealand	0.1	0.5	0.1	0.5	1
**Standard matrix (15 Ma - Root)**					
	East Asia	Australia	New Guinea	Pacific	New Zealand
East Asia	1	0.5	0	0.1	0.1
Australia	0.5	1	0	0.5	0.5
New Guinea	0	0	1	0	0
Pacific	0.1	0.5	0	1	0.5
New Zealand	0.1	0.5	0	0.5	1
**Relaxed matrix (Present to Root)**					
	East Asia	Australia	New Guinea	Pacific	New Zealand
East Asia	1	0.5	1	0.1	0.1
Australia	0.5	1	1	0.5	0.5
New Guinea	1	1	1	1	0.1
Pacific	0.1	0.5	1	1	0.5
New Zealand	0.1	0.5	0.1	0.5	1

Table S6. Marginal probabilities for the model selection approach Bayesian phylogenetic analyzes using the Dytiscidae dataset. Higher values mean better fit.

Table S6. Marginal probabilities for the model selection approach Bayesian phylogenetic analyzes using the Dytiscidae dataset. Higher values mean better fit.

Calibrations	Clock	Model	Path sampling	Stepping-Stone sampling
**Lognormal**	**Strict**	Best models	-356,787.13	-356,786.09
	**Strict**	Simpler model	-356,881.07	-356,879.77
	**Lognormal**	Best models	-353,496.13	-353,494.56
	**Lognormal**	Simpler model	-353,586.92	-353,586.33
**Exponential**	**Strict**	Best models	-356,794.10	-356,792.77
	**Strict**	Simpler model	-356,880.41	-356,879.25
	**Lognormal**	Best models	-353,493.64	-353,493.45
	**Lognormal**	Simpler model	-353,570.83	-353,570.47

Table S7. Prior selection for the Bayesian phylogenetic analyzes in the *Limbodessus* dataset. Different configurations for the partition were used: 1) All loci were analyzed together in PartitionFinder2 and 2) UCEs and the remaining loci were analyzed separately in PartitionFinder2.

Table S7. Prior selection for the Bayesian phylogenetic analyzes in the *Limbodessus* dataset. Different configuration for the partition were used: 1) All loci were analyzed together in PartitionFinder2 and 2) UCEs and the remaining loci were analyzed separately in PartitionFinder2.

Configuration	Clock	Model	Path sampling	Stepping-Stone sampling
**1**	**Strict**	Best models	-695,849.37	-695,849.51
	**Strict**	Simpler model	-696,206.22	-696,205.54
	**Lognormal**	Best models	-688,217.11	-688,223.53
	**Lognormal**	Simpler model	-688,537.84	-688,543.40
**2**	**Strict**	Best models	-696,345.24	-696,347.44
	**Strict**	Simpler model	-696,688.78	-696,687.92
	**Lognormal**	Best models	-688,977.87	-688,993.97
	**Lognormal**	Simpler model	-689,719.89	-689,726.40

Table S8. Hypotheses and model comparison for the biogeographical reconstruction.

Table S8. Hypotheses and model comparison for the biogeographical reconstruction.

Hypotheses	Model	LnL	# parameters	d	e	j	*w*	AICc	AICc weight	Root
M0 Unguided	DEC	-62.18	2	0.0025	0.001	0	-	128.5	0.69	BCDE
	DEC + *J*	-61.91	3	0.0025	0.001	0.0001	-	130.1	0.31	BCDE
	DIVALIKE	-71.97	2	0.0045	1.00E-12	0	-	148.1	3.90E-05	BC
	DIVALIKE + *J*	-71.76	3	0.0045	1.00E-12	0.0001	-	149.8	1.60E-05	BC
	BAYAREALIKE	-92.75	2	0.0034	0.022	0	-	189.6	3.70E-14	BC
	BAYAREALIKE + *J*	-67.48	3	0.0011	1.00E-07	0.015	-	141.2	0.0012	C
M1 Standard origin of New Guinea	DEC	-61.82	2	0.0039	0.0016	0	-	127.8	0.0028	BCDE
	DEC + *J*	-54.88	3	1.00E-12	1.00E-12	0.017	-	116	1	ABCD
	DIVALIKE	-71.06	2	0.007	1.00E-12	0	-	146.3	2.70E-07	BC
	DIVALIKE + *J*	-64.38	3	0.0026	1.00E-12	0.017	-	135	7.50E-05	BC
	BAYAREALIKE	-92.82	2	0.0058	0.021	0	-	189.8	9.70E-17	BC
	BAYAREALIKE + *J*	-68.88	3	0.0017	1.00E-07	0.023	-	144	8.30E-07	C
M2 Standard origin of New Guinea plus *w* parameter	DEC	-61.52	3	0.0055	0.0017	0	2.34	129.3	0.0037	BCDE
	DEC + *J*	-54.83	4	1.00E-12	1.00E-12	0.011	0.011	118.1	1	ABCD
	DIVALIKE	-70.45	3	0.01	1.00E-12	0	2.45	147.2	4.90E-07	BC
	DIVALIKE + *J*	-64.33	4	0.0022	1.00E-12	0.016	0.67	137.1	7.40E-05	BC
	BAYAREALIKE	-92.7	3	0.0048	0.021	0	0.56	191.7	1.10E-16	BC
	BAYAREALIKE + *J*	-67.48	4	0.0011	1.00E-07	0.015	0	143.4	3.20E-06	C
M3 Relaxed origin of New Guinea	DEC	-61.43	2	0.0039	0.0011	0	-	127	0.71	BCDE
	DEC + *J*	-61.25	3	0.0039	0.0011	0.0001	-	128.8	0.29	BCDE
	DIVALIKE	-70.97	2	0.007	1.00E-12	0	-	146.1	5.10E-05	BC
	DIVALIKE + *J*	-70.85	3	0.007	1.00E-12	0.0001	-	148	2.00E-05	BC
	BAYAREALIKE	-92.66	2	0.006	0.02	0	-	189.5	1.90E-14	BC
	BAYAREALIKE + *J*	-67.36	3	0.0017	1.00E-07	0.023	-	141	0.0006	C
M4 Relaxed origin of New Guinea plus *w* parameter	DEC	-61.09	3	0.0057	0.001	0	2.31	128.4	0.0056	BCDE
	DEC + *J*	-54.83	4	1.00E-12	1.00E-12	0.011	0.011	118.1	0.99	ABCD
	DIVALIKE	-70.34	3	0.011	1.00E-12	0	2.62	146.9	5.40E-07	BC
	DIVALIKE + *J*	-64.59	4	0.0017	1.00E-12	0.012	0.018	137.6	5.70E-05	BC
	BAYAREALIKE	-94.04	3	0.0096	0.022	0	2.79	194.3	2.80E-17	BC
	BAYAREALIKE + *J*	-67.23	4	0.0015	1.00E-07	0.019	0.57	142.9	4.10E-06	C

Supplementary references

1. Villastrigo, A., Deharveng, L. & Balke, M. New Caledonia's enigmatic terrestrial diving beetle *Typhlodessus monteithi* is a derived species of *Paroster*. *Zoologica Scripta* **52**, 176–183 (2023).
2. Prjibelski, A., Antipov, D., Meleshko, D., Lapidus, A. & Korobeynikov, A. Using SPAdes De Novo Assembler. *Curr. Protoc. Bioinformatics* **70**, 1–29 (2020).
3. Laetsch, D. R. & Blaxter, M. L. BlobTools: Interrogation of genome assemblies. *F1000Res.* **6**, 1287 (2017).
4. Faircloth, B. C. PHYLUCE is a software package for the analysis of conserved genomic loci. *Bioinformatics* **32**, 786–788 (2016).
5. Gustafson, G. T. *et al.* Ultraconserved element (UCE) probe set design: Base genome and initial design parameters critical for optimization. *Ecol. Evol.* **9**, 6933–6948 (2019).
6. Minh, B. Q. *et al.* IQ-TREE 2: New Models and Efficient Methods for Phylogenetic Inference in the Genomic Era. *Mol. Biol. Evol.* **37**, 1530–1534 (2020).
7. Baca, S. M., Gustafson, G. T., Alexander, A. M., Gough, H. M. & Toussaint, E. F. A. Integrative phylogenomics reveals a Permian origin of Adephaga beetles. *Syst. Entomol.* **46**, 968–990 (2021).
8. Vasilikopoulos, A. *et al.* Phylogenomic analyses clarify the pattern of evolution of Adephaga (Coleoptera) and highlight phylogenetic artefacts due to model misspecification and excessive data trimming. *Syst. Entomol.* **46**, 991–1018 (2021).
9. Madden, T. L. *et al.* BLAST+: architecture and applications. *BMC Bioinformatics* **10**, 421 (2009).
10. Langille, B. L. *et al.* Parallel decay of vision genes in subterranean water beetles. *Mol. Phylogenet. Evol.* **173**, 107522 (2022).
11. Talavera, G., Lukhtanov, V., Pierce, N. E. & Vila, R. DNA Barcodes Combined with Multilocus Data of Representative Taxa Can Generate Reliable Higher-Level Phylogenies. *Syst. Biol.* **71**, 382–395 (2022).
12. Duchêne, D. A., Mather, N., Van Der Wal, C. & Ho, S. Y. W. Excluding loci with substitution saturation improves inferences from phylogenomic data. *Syst. Biol.* **71**, 676–689 (2022).
13. Mongiardino Koch, N. Phylogenomic Subsampling and the Search for Phylogenetically Reliable Loci. *Mol. Biol. Evol.* **38**, 4025–4038 (2021).
14. Kalyaanamoorthy, S., Minh, B. Q., Wong, T. K. F., von Haeseler, A. & Jermiin, L. S. ModelFinder: fast model selection for accurate phylogenetic estimates. *Nat. Methods* **14**, 587–589 (2017).
15. Lanfear, R., Frandsen, P. B., Wright, A. M., Senfeld, T. & Calcott, B. PartitionFinder 2: New methods for selecting partitioned models of evolution for molecular and morphological phylogenetic analyses. *Mol. Biol. Evol.* **34**, 772–773 (2017).
16. Suchard, M. A. *et al.* Bayesian phylogenetic and phylodynamic data integration using BEAST 1.10. *Virus Evolution* **4**, 1–5 (2018).
17. Nilsson, A. N. & Hájek, J. A world catalogue of the family Dytiscidae, or the Diving beetles (Coleoptera, Adephaga). https://waterbeetles.eu/documents/W_CAT_Dytiscidae_2024_new.pdf (2024).
18. Baele, G. *et al.* Improving the accuracy of demographic and molecular clock model comparison while accommodating phylogenetic uncertainty. *Mol. Biol. Evol.* **29**, 2157–2167 (2012).
19. Baele, G. & Lemey, P. Bayesian evolutionary model testing in the phylogenomics era: matching model complexity with computational efficiency. *Bioinformatics* **29**, 1970–1979 (2013).
20. Matzke, N. J. Probabilistic Historical Biogeography: New Models for Founder-Event Speciation, Imperfect Detection, and Fossils Allow Improved Accuracy and Model-Testing. (University of California, 2013).

21. Cloos, M. *et al.* Collisional delamination in New Guinea: The geotectonics of subducting slab breakoff. (2005).

22. Gold, D. P., White, L. T., Gunawan, I. & BouDagher-Fadel, M. K. Relative sea-level change in western New Guinea recorded by regional biostratigraphic data. *Mar. Pet. Geol.* **86**, 1133–1158 (2017).

23. Ree, R. H. & Sanmartín, I. Conceptual and statistical problems with the DEC +J model of founder-event speciation and its comparison with DEC via model selection. *J. Biogeogr.* **45**, 741–749 (2018).

24. Revell, L. J. phytools: An R package for phylogenetic comparative biology (and other things). *Methods Ecol. Evol.* **3**, 217–223 (2012).

25. Leys, R., Watts, C. H. S., Cooper, S. J. B. & Humphreys, W. F. Evolution of subterranean diving beetles (Coleoptera: Dytiscidae: Hydroporini, Bidessini) in the arid zone of Australia. *Evolution* **57**, 2819–2834 (2003).

26. Leijs, R. *et al.* Evolution of blind beetles in isolated aquifers: A test of alternative modes of speciation. *PLoS One* **7**, (2012).

27. Langille, B. L. *et al.* Evidence for speciation underground in diving beetles (Dytiscidae) from a subterranean archipelago. *Evolution* **75**, 166–175 (2021).

28. Cooper, S. J. B., Hinze, S., Leys, R., Watts, C. H. S. & Humphreys, W. F. Islands under the desert: molecular systematics and evolutionary origins of stygobiontic water beetles (Coleoptera: Dytiscidae) from central Western Australia. *Invertebr. Syst.* **16**, 589–598 (2002).

29. Watts, C. H. S. & Humphreys, W. F. Twenty-Six New Dytiscidae (Coleoptera) of the Genera *Limbodessus* Guignot and *Nirripirti* Watts & Humphreys, from Underground Waters in Australia. *Trans. R. Soc. S. Aust.* **130**, 123–185 (2006).

30. Jones, K. K., Cooper, S. J. B. & Seymour, R. S. Cutaneous respiration by diving beetles from underground aquifers of Western Australia (Coleoptera: Dytiscidae). *J. Exp. Biol.* **222**, 1–13 (2019).

31. Jones, K. K. *et al.* The critical thermal maximum of diving beetles (Coleoptera: Dytiscidae): a comparison of subterranean and surface-dwelling species. *Curr. Res. Insect Sci.* **1**, 100019 (2021).

32. Tierney, S. M. *et al.* Opsin transcripts of predatory diving beetles: a comparison of surface and subterranean photic niches. *Royal Society open science* **2**, 140386 (2015).

33. Katoh, K. & Standley, D. M. MAFFT multiple sequence alignment software version 7: Improvements in performance and usability. *Mol. Biol. Evol.* **30**, 772–780 (2013).

34. Kearse, M. *et al.* Geneious Basic: An integrated and extendable desktop software platform for the organization and analysis of sequence data. *Bioinformatics* **28**, 1647–1649 (2012).

35. Phillips, S. J., Anderson, R. P., Dudík, M., Schapire, R. E. & Blair, M. E. Opening the black box: an open-source release of Maxent. *Ecography* **40**, 887–893 (2017).

36. Booth, T. H., Nix, H. A., Busby, J. R. & Hutchinson, M. F. BIOCLIM: the first species distribution modelling package, its early applications and relevance to most current MAXENT studies. *Diversity and Distributions* **20**, 1–9 (2014).

37. Balke, M. & Ribera, I. Jumping across Wallace's line: *Allodessus* Guignot and *Limbodessus* Guignot revisited (Coleoptera: Dytiscidae, Bidessini) based on molecular-phylogenetic and morphological data. *Aust. J. Entomol.* **43**, 114–128 (2004).

38. Waters, J. M., Emerson, B. C., Arribas, P. & McCulloch, G. A. Dispersal Reduction: Causes, Genomic Mechanisms, and Evolutionary Consequences. *Trends Ecol. Evol.* **35**, 512–522 (2020).

39. Culver, D. C., Kowalko, J. E. & Pipan, T. Natural selection versus neutral mutation in the evolution of subterranean life: A false dichotomy? *Frontiers in Ecology and Evolution* **11**, (2023).
